# Author Correction: Multistate structures in a hydrogen-bonded polycatenation non-covalent organic framework with diverse resistive switching behaviors

**DOI:** 10.1038/s41467-024-45253-8

**Published:** 2024-01-29

**Authors:** Shimin Chen, Yan Ju, Yisi Yang, Fahui Xiang, Zizhu Yao, Hao Zhang, Yunbin Li, Yongfan Zhang, Shengchang Xiang, Banglin Chen, Zhangjing Zhang

**Affiliations:** 1 Fujian Provincial Key Laboratory of Polymer Materials, College of Materials Science and Engineering, Fujian Normal University, Fuzhou, 350007 Fujian China; 2 College of Chemistry, Fuzhou University, Fuzhou, 350108 China

**Keywords:** Self-assembly, Self-assembly, Information storage

Correction to: *Nature Communications* 10.1038/s41467-023-44214-x, published online 5 January 2024

In this article the grant number 22271046 relating to Natural Science Foundation of China was omitted. The original article has been corrected.

