# Supplementary material for: Multistate structures in a hydrogen-bonded polycatenation non-covalent organic framework with diverse resistive switching behaviors
Source: Nat Commun. 2024 Jan 5;15:298. doi: 10.1038/s41467-023-44214-x (PMC10770064; doi:10.1038/s41467-023-44214-x)
Supplement: Supplementary file 1 — Supplementary Information [file 41467_2023_44214_MOESM1_ESM.pdf]

# Supplementary Information

## Multistate structures in a hydrogen-bonded polycatenation non-covalent organic framework with diverse resistive switching behaviors

Shimin Chen[1,#], Yan Ju[1,#], Yisi Yang[1], Fahui Xiang[1], Zizhu Yao[1], Hao Zhang[1], Yunbin Li[1], Yongfan Zhang[2], Shengchang Xiang[1], Banglin Chen[1], Zhangjing Zhang[1] ✉

**This PDF file includes:**

Supplementary Figures 1 to 25
Supplementary Tables 1 to 5
Supplementary References

# Table of Contents


| | |
|---|---|
| **Supplementary Figure 1** | The *trans* and *cis* conformations of $[H_2L]Cl_2$. |
| **Supplementary Figure 2** | The TGA curve for HOF-FJU-52. |
| **Supplementary Figure 3** | Structure of HOF-FJU-52 single crystal. |
| **Supplementary Figure 4** | A packing diagram of HOF-FJU-52 showing the cage surfaces. |
| **Supplementary Figure 5** | Water vapor sorption isotherm of HOF-FJU-52a. |
| **Supplementary Figure 6** | The powder X-ray diffraction patterns of HOF-FJU-52. |
| **Supplementary Figure 7** | The shrinkage rate of the cell parameters *b* of the HOF structures. |
| **Supplementary Figure 8** | Crystal Structure of HOF-FJU-52-200K. |
| **Supplementary Figure 9** | Structure changes upon altering the temperatures. |
| **Supplementary Figure 10** | VTPXRD patterns of HOF-FJU-52 in vacuum condition. |
| **Supplementary Figure 11** | Electrical performance of HOF-FJU-52 single crystal along the *a* and *c* axes. |
| **Supplementary Figure 12** | The comparison of the set voltage and ON/OFF ratio between HOF-FJU-52 and some representative RS materials. |
| **Supplementary Figure 13** | RS behaviors of the HOF-FJU-52 single crystal device upon the temperatures, humidities, and solvents. |
| **Supplementary Figure 14** | RS characteristics of Ag/HOF-FJU-52/Ag single crystal device under ultralow $I_{CC}$ down to 110 and 11 pA. |
| **Supplementary Figure 15** | Current-voltage curves of the HOF-FJU-52a device in vacuum condition at 353 K. |
| **Supplementary Figure 16** | Pulse measurement of HOF-FJU-52a under vacuum at 353 K. |
| **Supplementary Figure 17** | Schematic diagram for the RRAM behaviors of our HOF single crystal device along the *b* axis upon cooling. |
| **Supplementary Figure 18** | Schematic diagram for the WORM behaviors of our HOF single crystal device along the *b* axis upon heating. |
| **Supplementary Figure 19** | The recovery of RRAM behavior of the single crystal device for 5 hours at room temperature. |
| **Supplementary Figure 20** | ATR-IR spectra of eight HOF-FJU-52 single crystals before and after the voltage stimulus at 0.6 V for 20 minutes. |
| **Supplementary Figure 21** | The changes of dihedral angle and infinite π-π stacking column under dc voltages applied on HOF-FJU-52 along the *b* axis. |
| **Supplementary Figure 22** | XPS core-level spectra of O1s of $H_2L$ and $H_8PTP$. |
| **Supplementary Figure 23** | The structure variations of HOF-FJU-52-100K along the *b* axis under the voltage stimulus. |
| **Supplementary Figure 24** | The microscopic image of the experimental setup. |
| **Supplementary Figure 25** | Electrical performance of the HOF-FJU-52 pellet device. |
| **Supplementary Table 1** | Comparison with state of the art MOF/COF-based RS materials. |
| **Supplementary Table 2** | Comparison with state of the art organic RS materials. |
| **Supplementary Table 3** | Comparison with state of the art inorganic RS materials. |
| **Supplementary Table 4** | Crystal data and structure refinement for HOF-FJU-52 under voltage sweeping and different temperatures stimulus. |
| **Supplementary Table 5** | Crystal data and structure refinement for HOF-FJU-52-100K under voltage sweeping. |

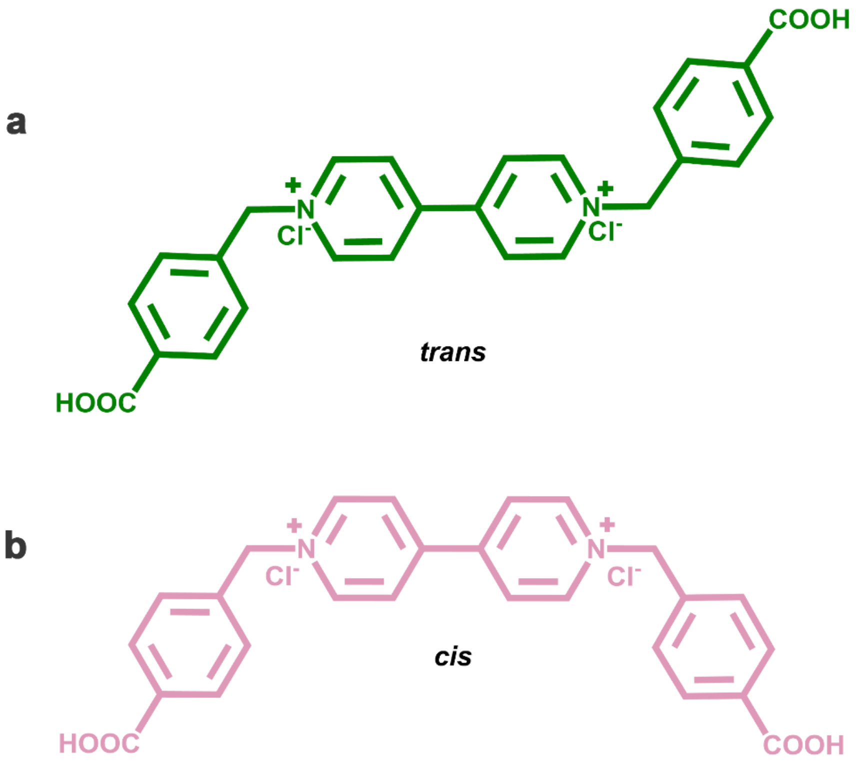


**Supplementary Figure 1.** The *trans* (**a**) and *cis* (**b**) conformations of [$H_2L$]$Cl_2$.

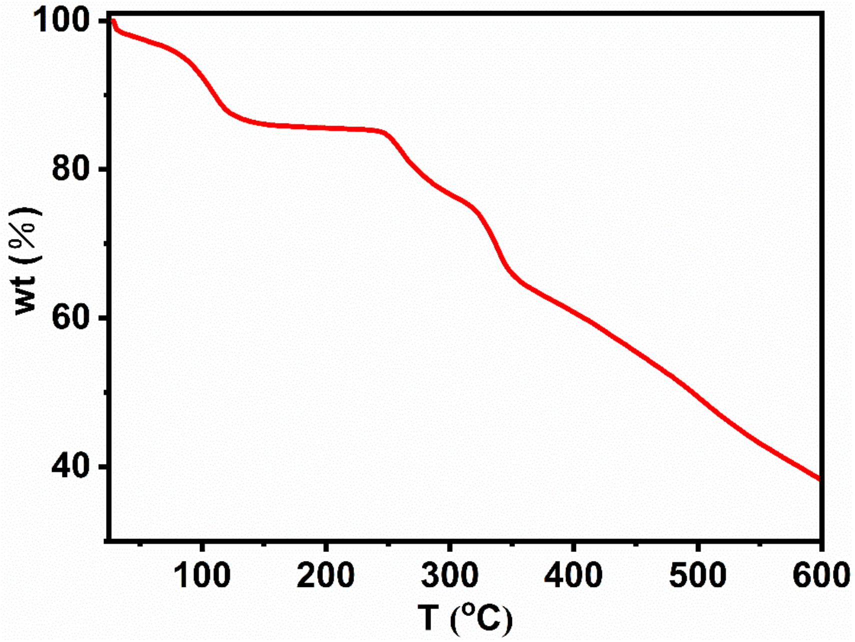


**Supplementary Figure 2.** The TGA curve for HOF-FJU-52 under $N_2$ atmosphere with heating rate of 10 °C min$^{-1}$.

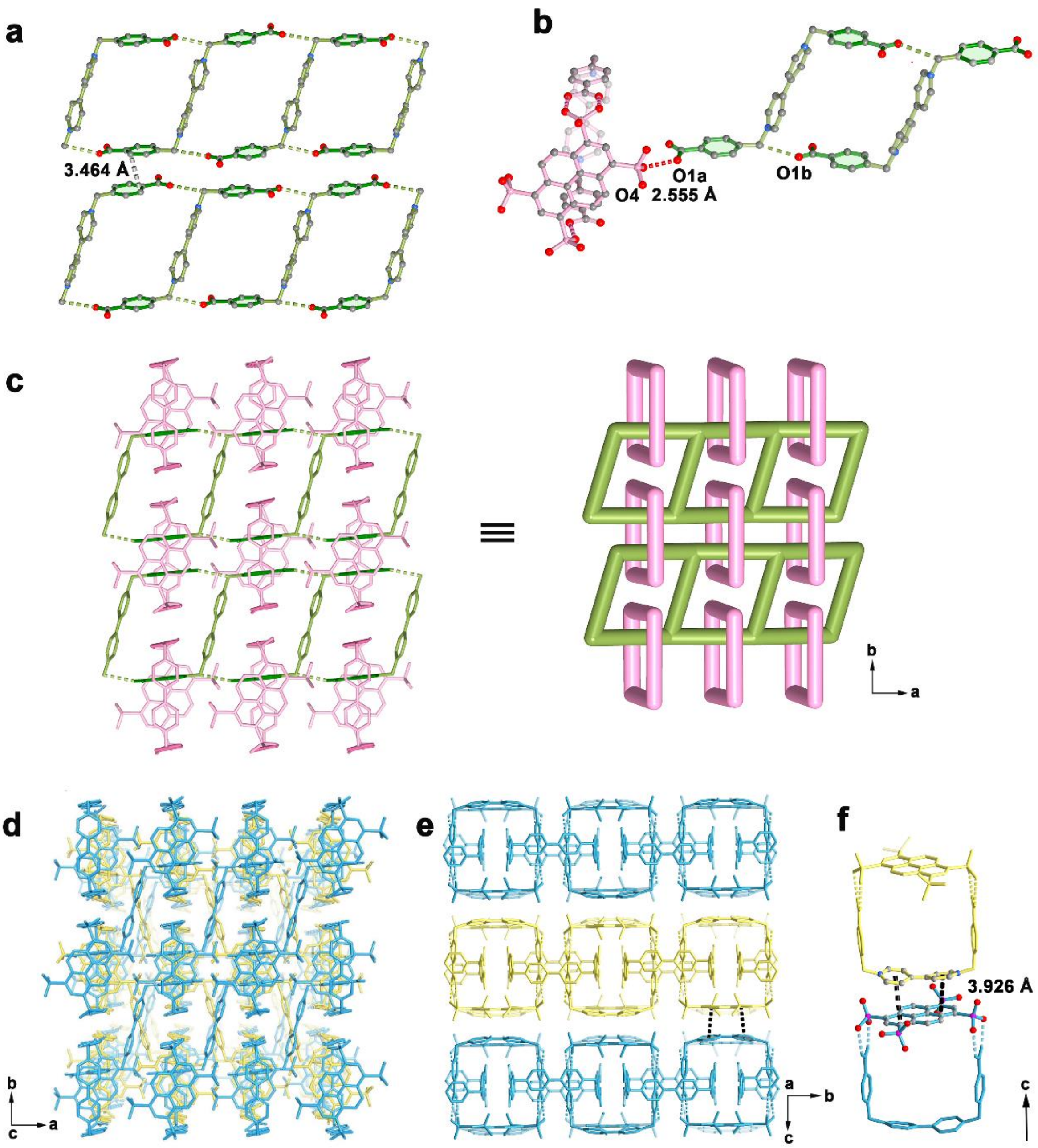


**Supplementary Figure 3.** Structure of HOF-FJU-52 single crystal. **a** The π-π interaction between one-dimensional ladder chain. **b** The hydrogen bond interaction (O1-H1···O4) between ladder chain and molecular loop. **c** The polycatenation sheet and its simplification along *ab* plane (green dashed lines: O1-H1···O4). **d** A packing diagram of HOF-FJU-52 showing the *ab* planes. **e** The π-π interactions between the 2D *ab* planes. **f** The π-π interaction between face-to-face planes of the two adjacent molecular loops. Guest molecules have been omitted for clarity.

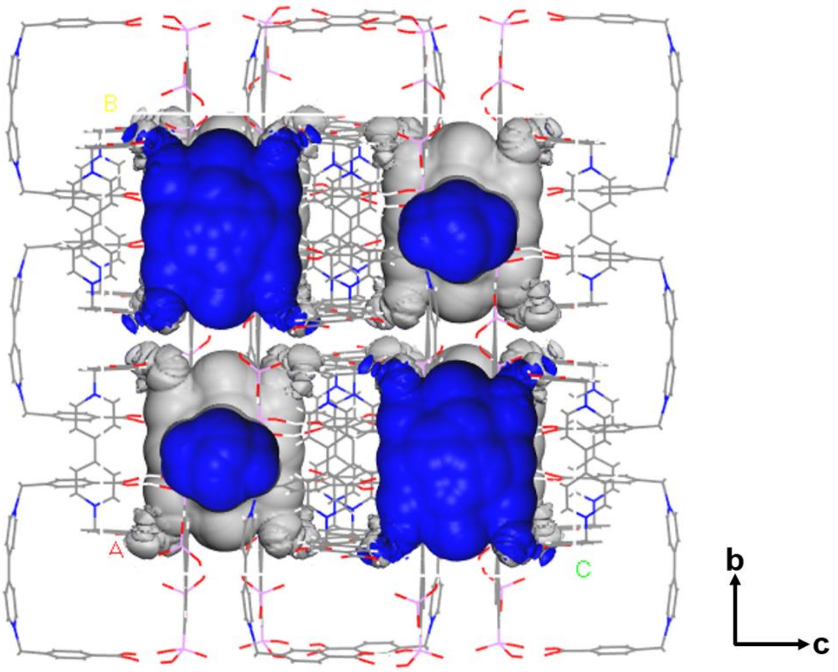


**Supplementary Figure 4.** A packing diagram of HOF-FJU-52 showing the cage surfaces highlighted as blue/gray (inner/outer) curved planes.

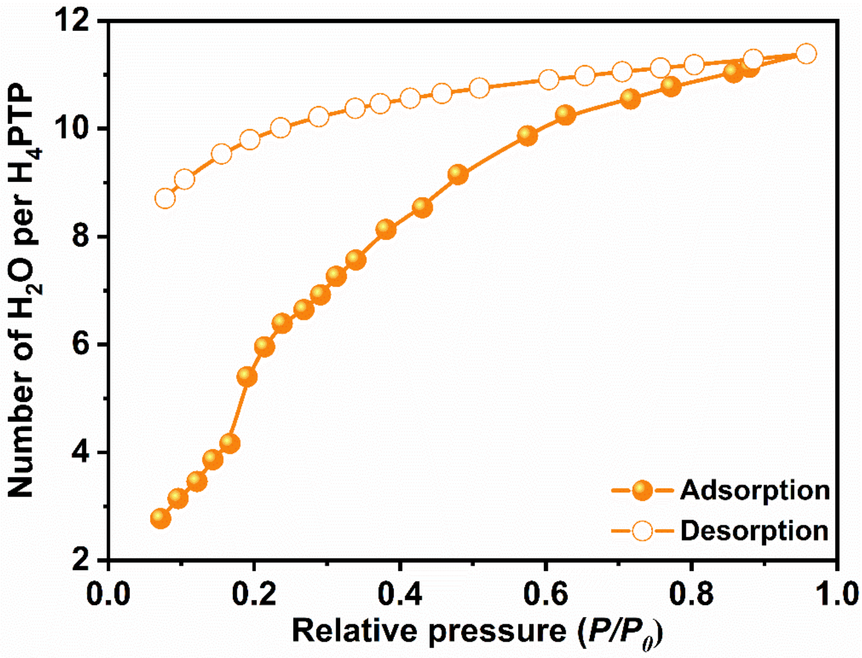


**Supplementary Figure 5.** Water vapor sorption isotherms of HOF-FJU-52a (294K). The orange filled and open symbols denote adsorption and desorption processes, respectively.

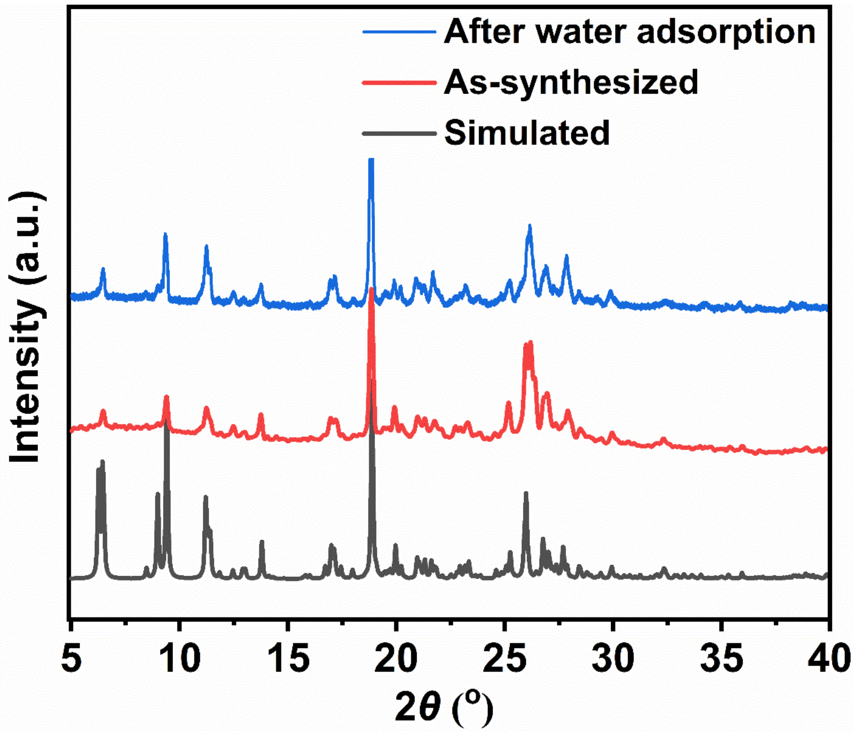


**Supplementary Figure 6.** The powder X-ray diffraction patterns of HOF-FJU-52.

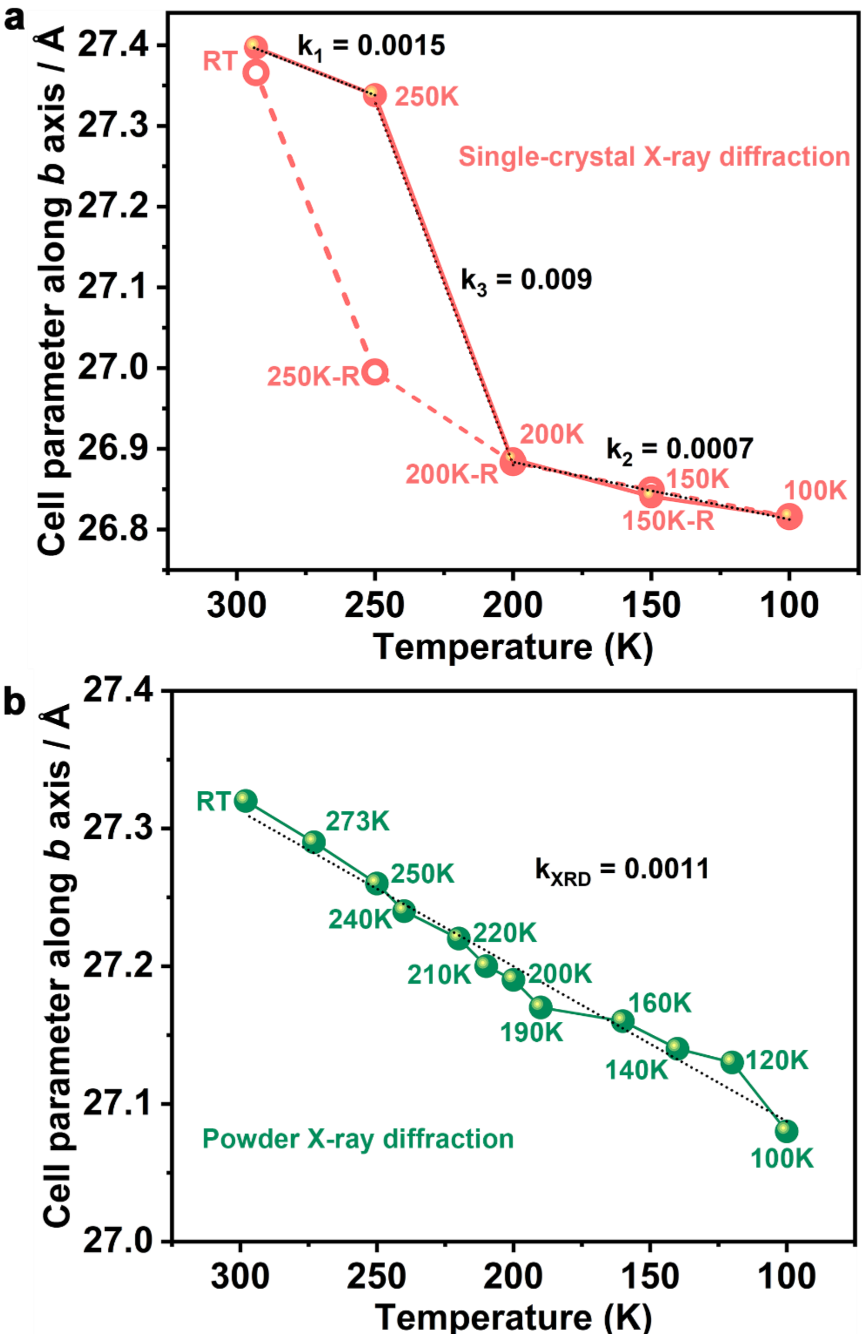


**Supplementary Figure 7.** The shrinkage rate of the cell parameters *b* of the HOF structures. The shrinkage rate calculated from the slope in the function of the cell parameters *b* with the temperatures from single crystal XRD data (orange color) (**a**) and the VTPXRD data (green color) (**b**) of our HOF single crystal.

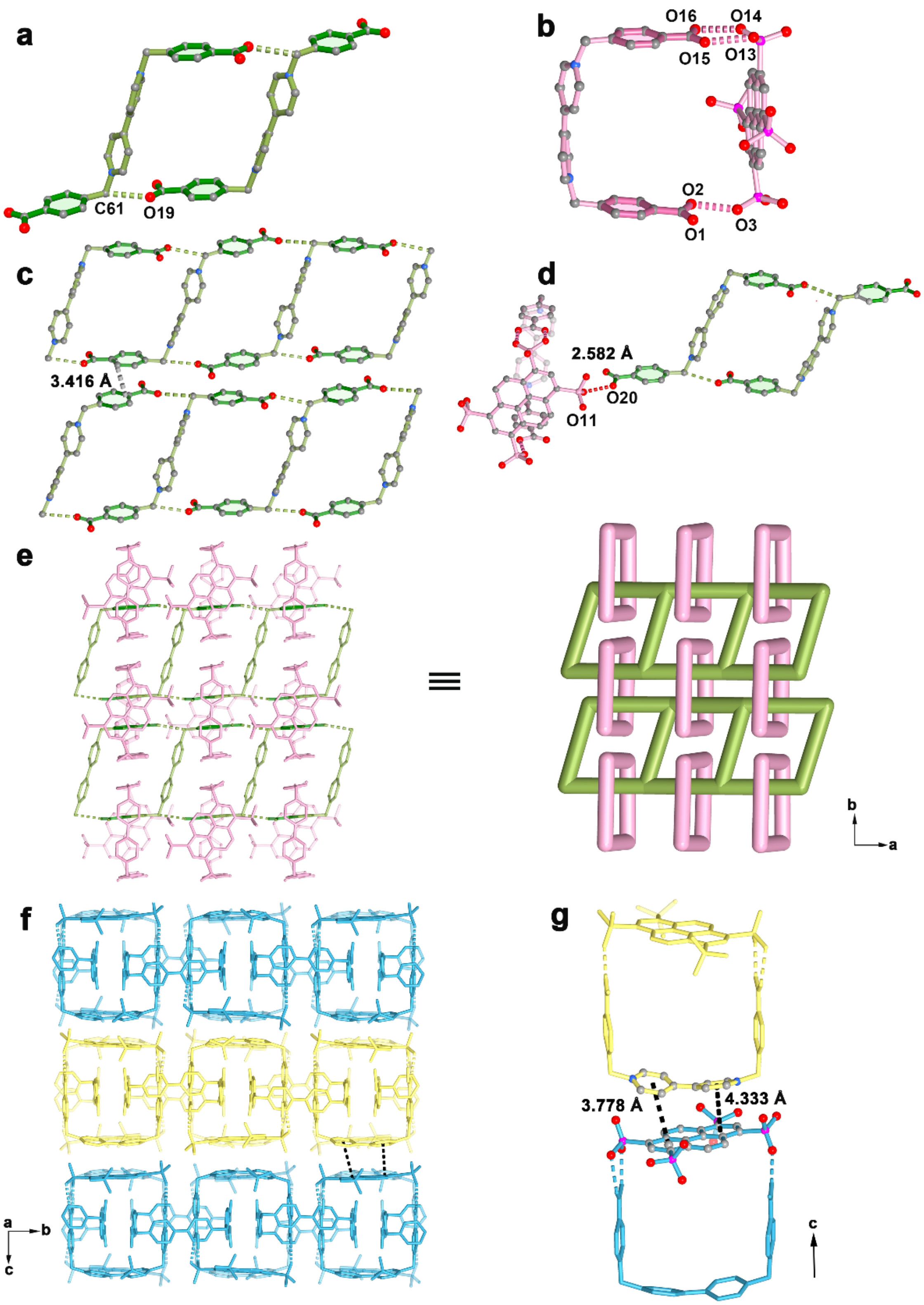


**Supplementary Figure 8.** Crystal structure of HOF-FJU-52-200 K. **a** Ladder chain $[(H_2L^1)_n]^{2n+}$. **b** Molecular loop $[(H_4L^2)(H_2L^1)]^{2-}$, half the hydrogen bond dimers are destructed. **c** The π-π interaction between one-dimensional ladder chain. **d** The hydrogen bond interaction (O20-H20···O11) between ladder chain and molecular loop. **e** The 0D + 1D → 2D polycatenated sheet (green dashed lines: O20-H20···O11). **f** A packing diagram of HOF-FJU-52-200 K showing the *bc* planes. **g** The π-π interaction between face-to-face planes of molecular loops. Guest water molecules and part of H atoms have been omitted for clarity.

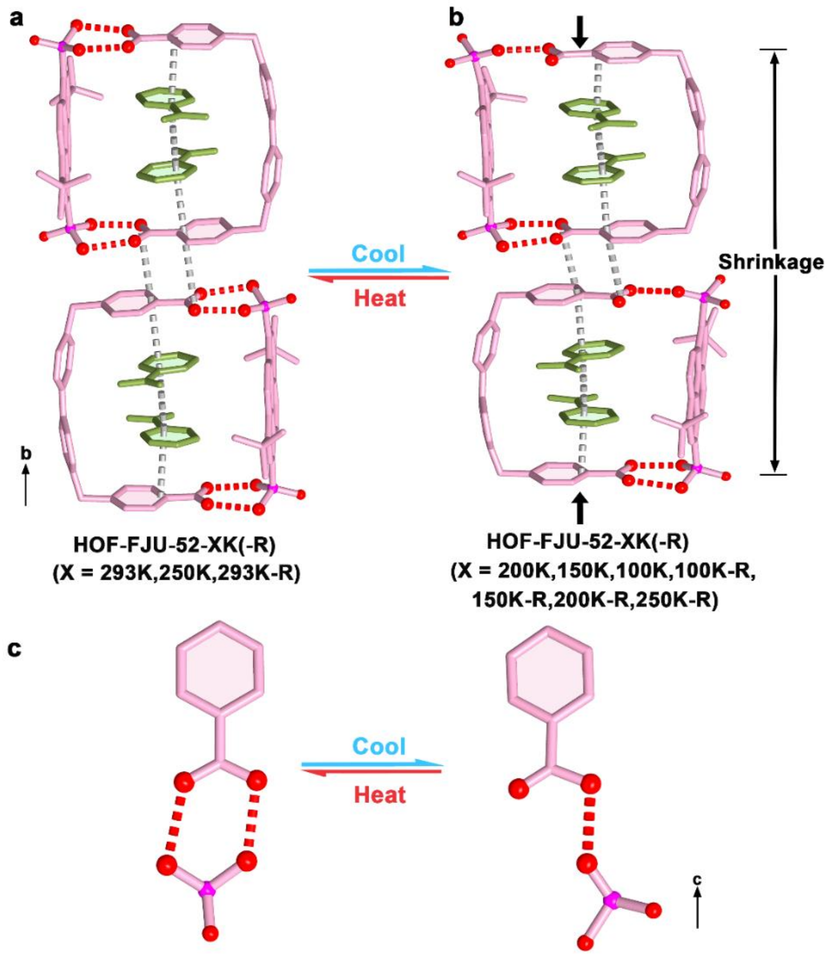


**Supplementary Figure 9.** Structure changes upon altering the temperatures. **a** and **b** The infinite π-π stacking column through staggered 4-carboxyphenyl groups. Upon cooling, half the hydrogen bond dimers are destructed (**b**). **c** Comparison between the hydrogen bond dimer (left) and the H bond monomer (right). The O-H···O hydrogen bonds are shown as the red dash lines.

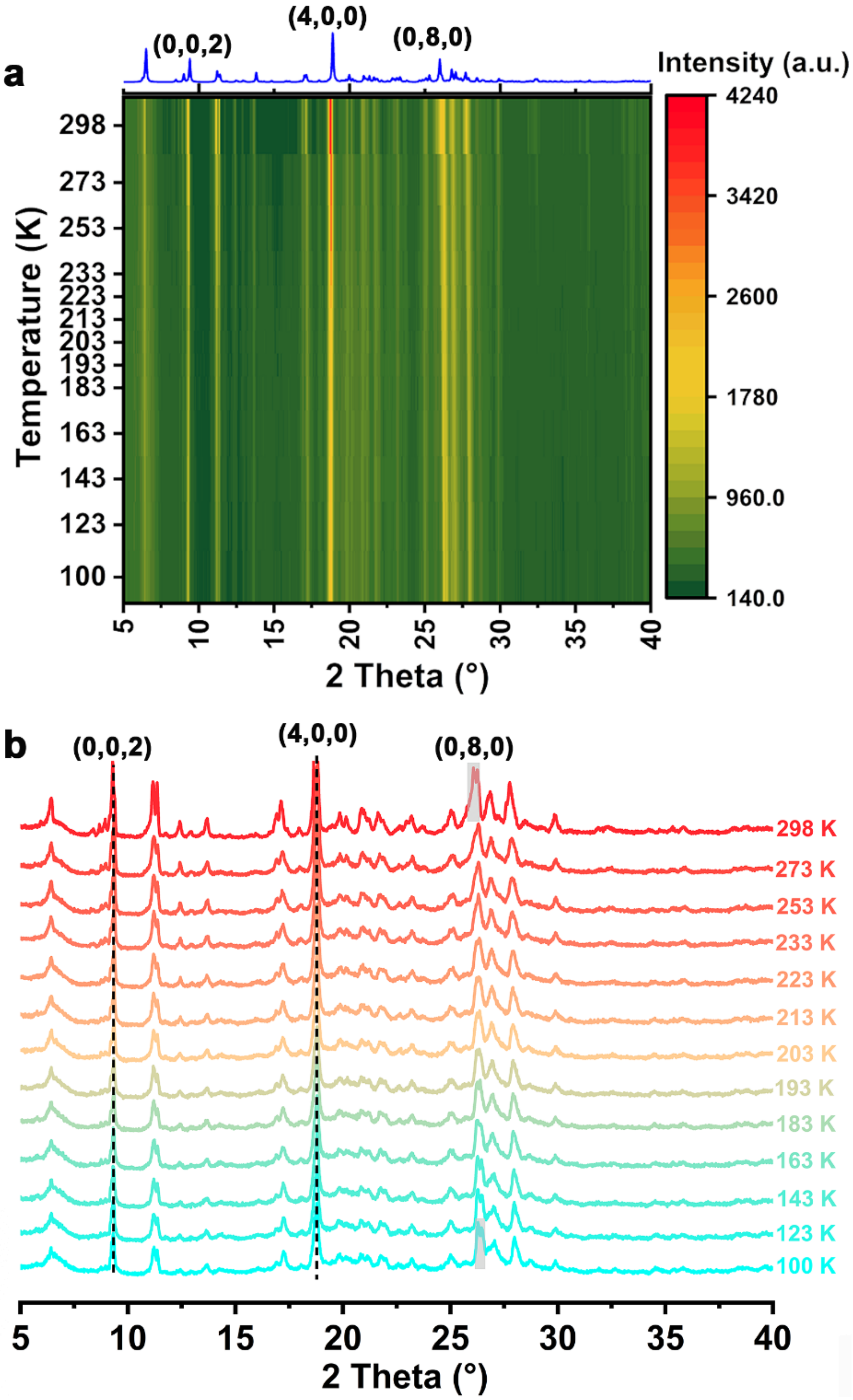


**Supplementary Figure 10.** The variable temperature powder X-ray diffraction (VTPXRD) pattern of HOF-FJU-52. **a** Contour plot of the VTPXRD pattern evolution (λ = 1.54056 Å) of HOF-FJU-52 in vacuum condition. **b** VTPXRD patterns of HOF-FJU-52 in vacuum condition.

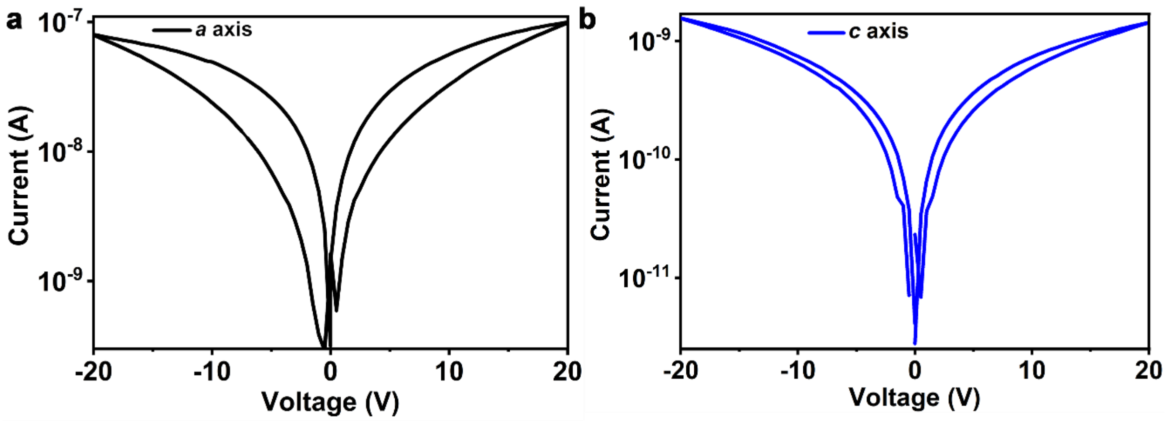


**Supplementary Figure 11.** Electrical performance of HOF-FJU-52 single crystal. The semilogarithmic plot of the room-temperature current-voltage (*I-V*) characteristics of the single crystal HOF-FJU-52 along the *a* (**a**) and *c* (**b**) axes.

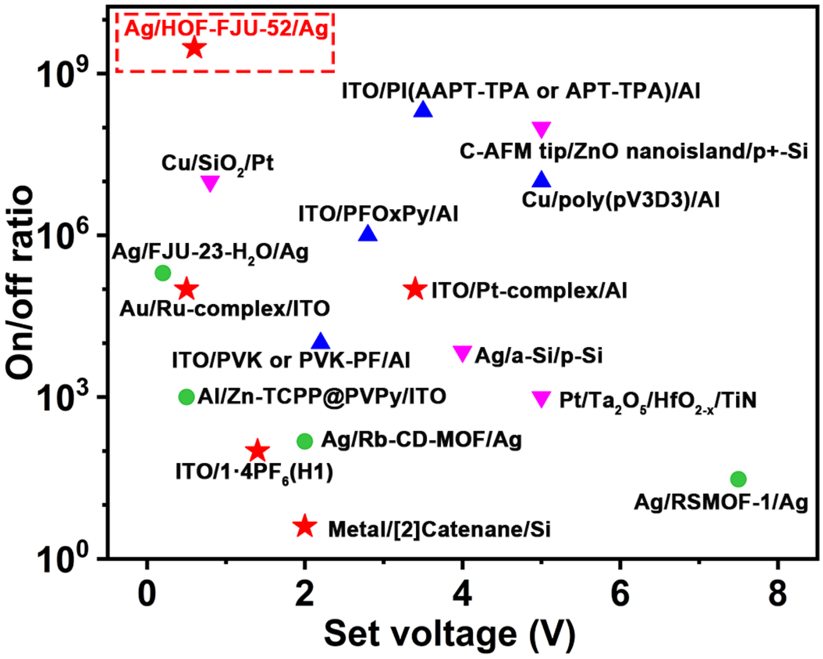


**Supplementary Figure 12.** The comparison of the set voltage and ON/OFF ratio between HOF-FJU-52 and some representative RS materials. As presented in tables S1-S3 (symbol code: red star = supramolecular materials, green circle = MOF materials, blue uptriangel= polymer materials, magenta downtriangel= inorganic materials).

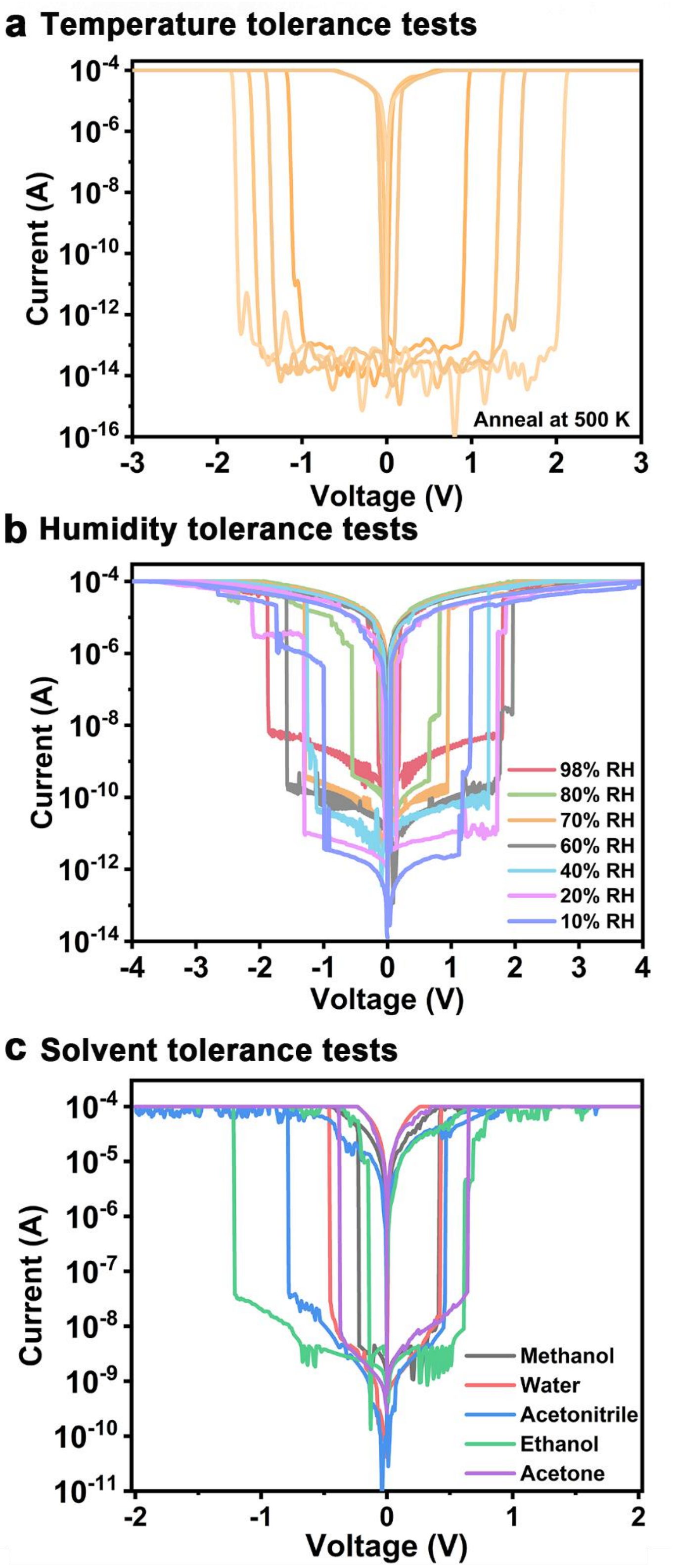


**Supplementary Figure 13.** RS behaviors of the HOF-FJU-52 single crystal device upon annealed at 500 K in vacuum condition (**a**), at 25 °C with different relative humidity (**b**), and soaked in various solvents (**c**).

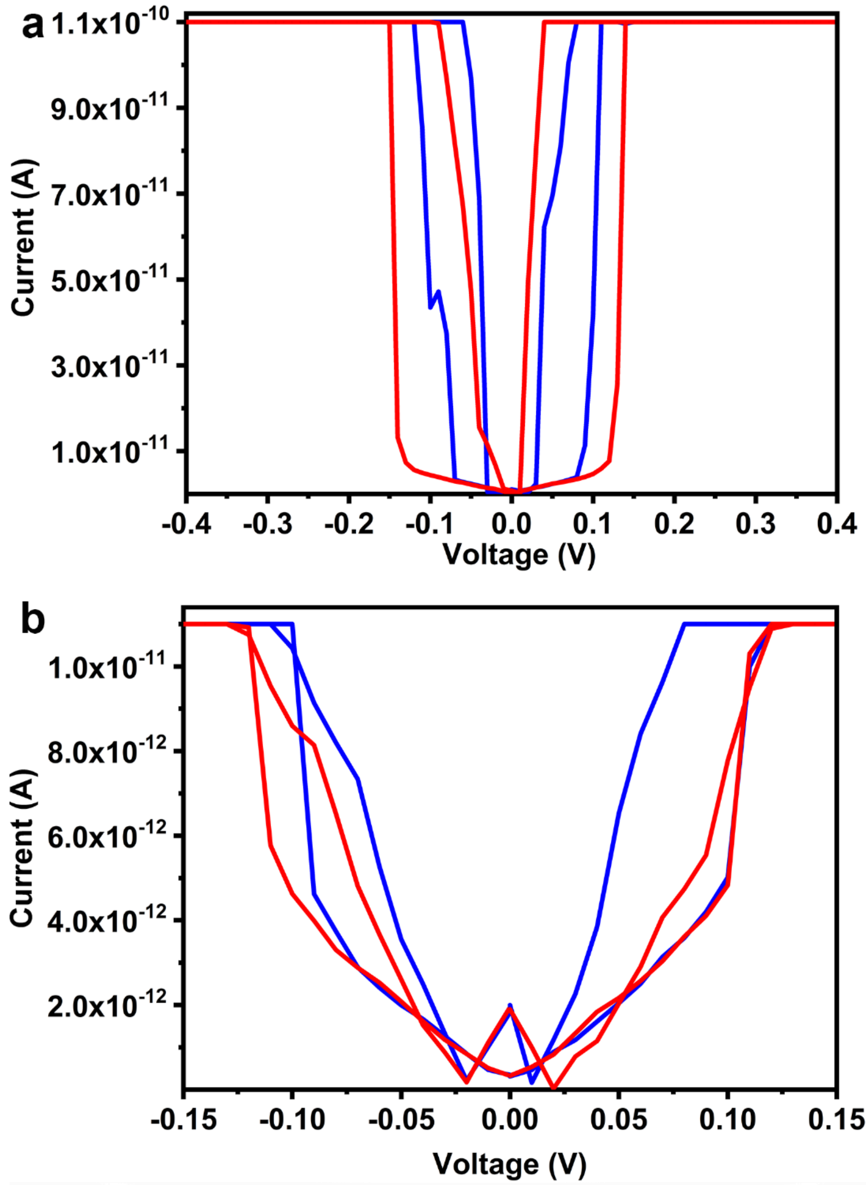


**Supplementary Figure 14.** RS characteristics of the Ag/HOF-FJU-52/Ag single crystal device under ultralow $I_{CC}$ down to 110 (**a**) and 11 pA (**b**).

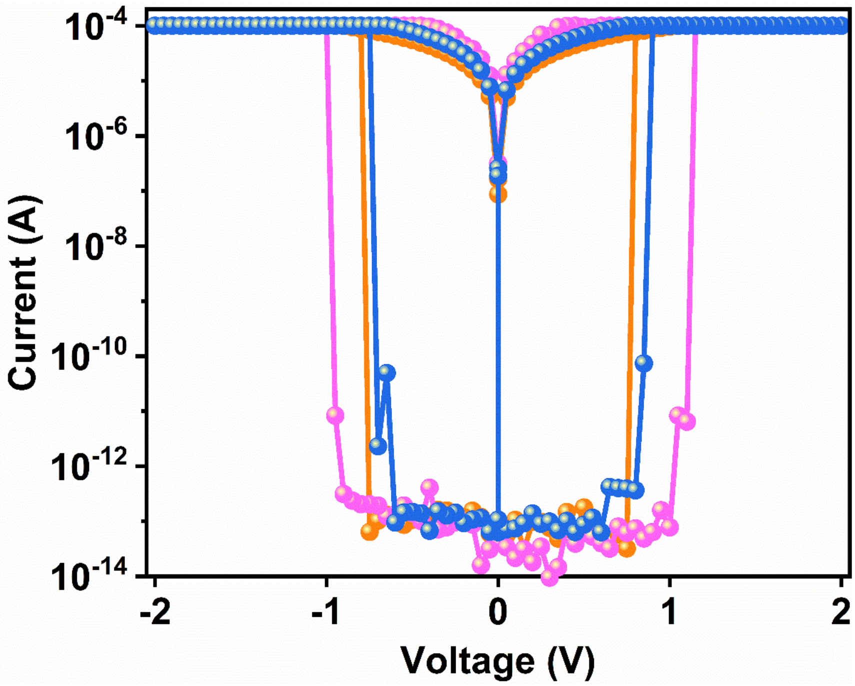

**Supplementary Figure 15.** Current-voltage curves of the HOF-FJU-52a device in vacuum condition at 353 K.

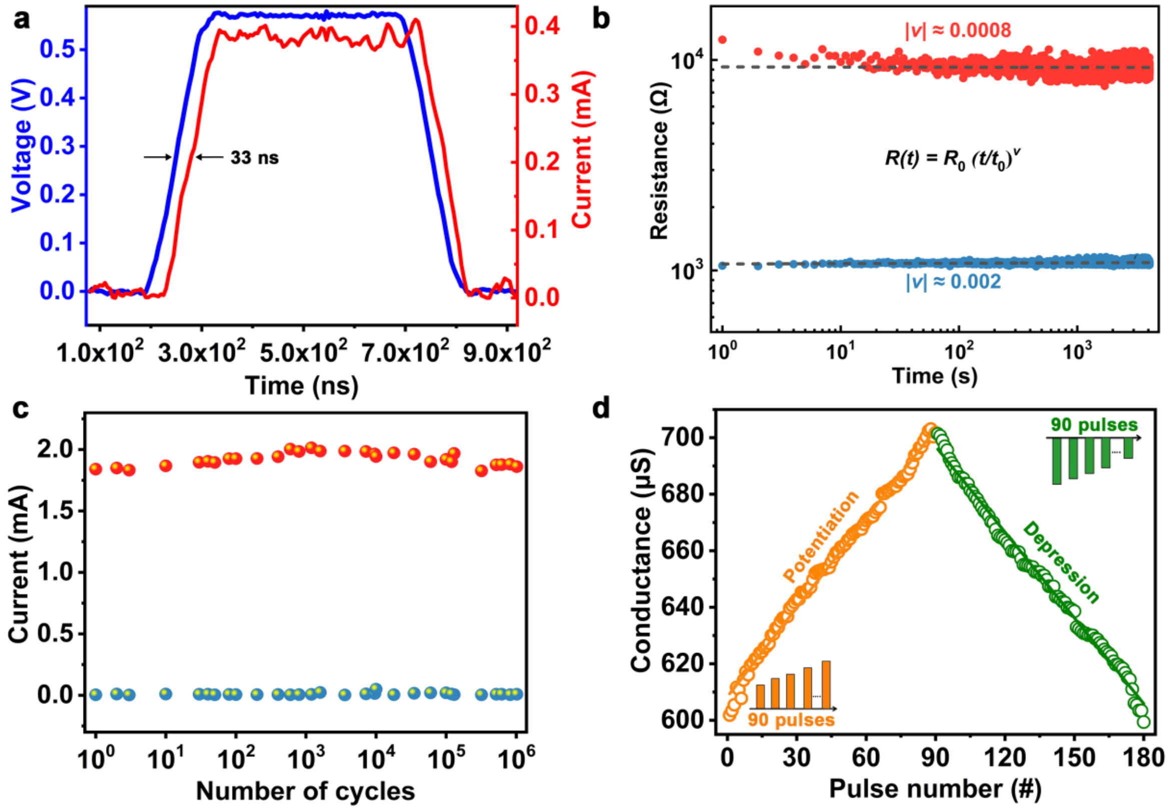


**Supplementary Figure 16.** Pulse measurement of HOF-FJU-52a under vacuum at 353 K. **a** Current response with a fast voltage pulse. **b** Ultralow resistance drift for the HRS and LRS state of the HOF-FJU-52a device, $R_0$ is the resistance at an arbitrary time $t_0$, and $t$ is the time after the last switching event. The programming was carried out with 500 ns voltage pulses of -0.1 V and 2.7 V to HRS and LRS, respectively. **c** Stable current values are found for pulse endurance test of the HOF-FJU-52a device consistently. The programming was carried out with 500 ns voltage pulses of -0.1 V and 2 V to RESET and SET, respectively. **d** Potentiation and depression process measured with stepwise voltage pulses. For potentiation, the pulse amplitude increases from 0.13 to 1.85 V with 20 mV steps; for depression, the pulse amplitude decreases from to 1.83 to 0.11 V with 20 mV steps (pulse width: 200 ns).

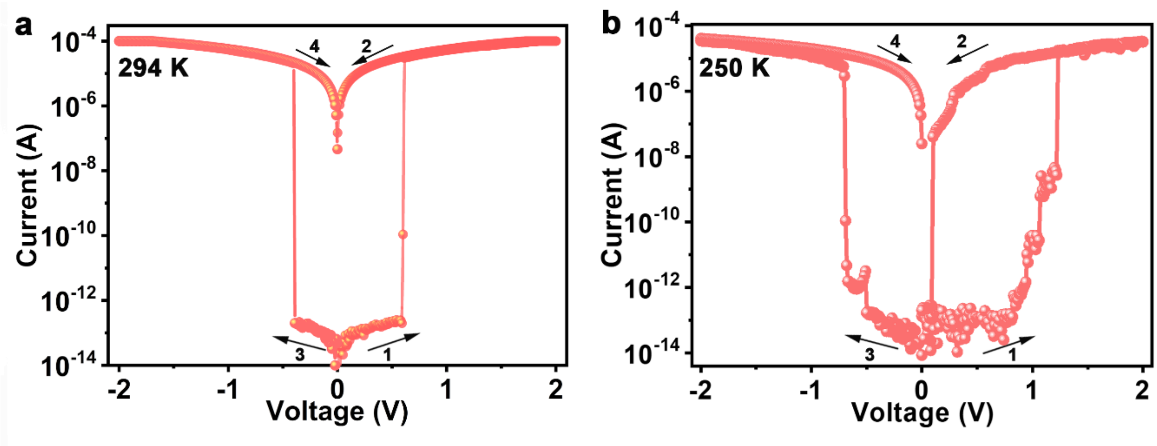


**Supplementary Figure 17.** Schematic diagram for the RRAM behavior of our HOF single crystal device along the *b* axis at 294 (**a**) and 250 K (**b**) upon cooling at an interval of 50 K.

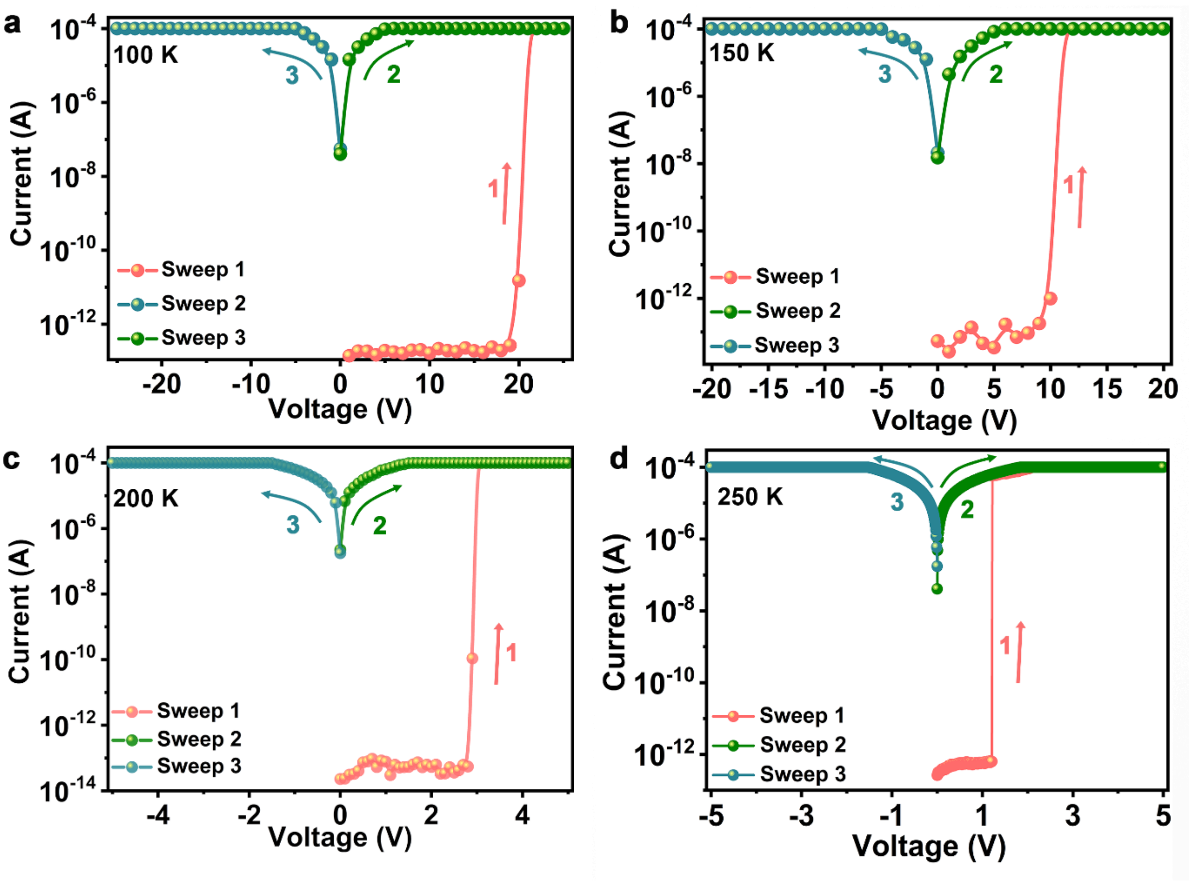


**Supplementary Figure 18.** Schematic diagram for the WORM behavior of our HOF single crystal device along the *b* axis at 100 K (**a**), 150 K (**b**), 200 K (**c**) and 250 K (**d**) upon heating at an interval of 50 K.

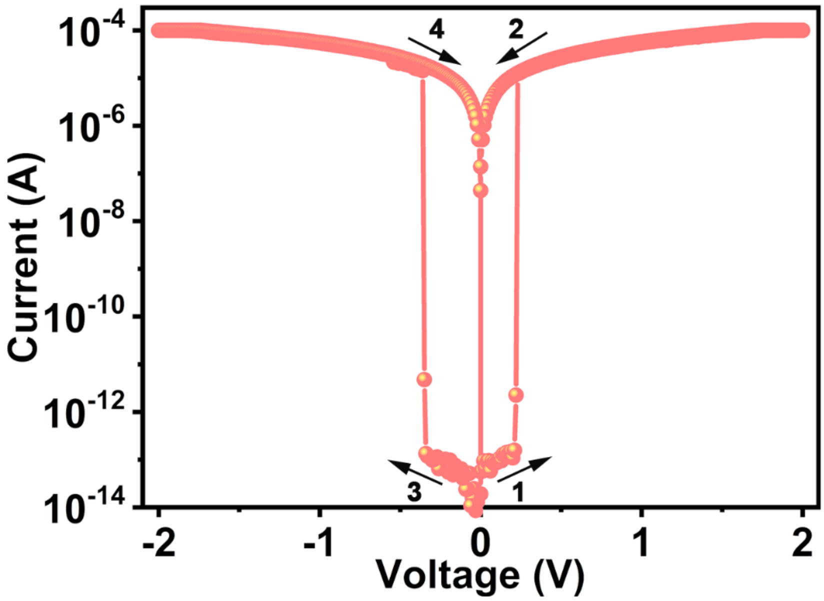


**Supplementary Figure 19.** The recovery of RRAM behavior of the single crystal device for 5 hours at room temperature.

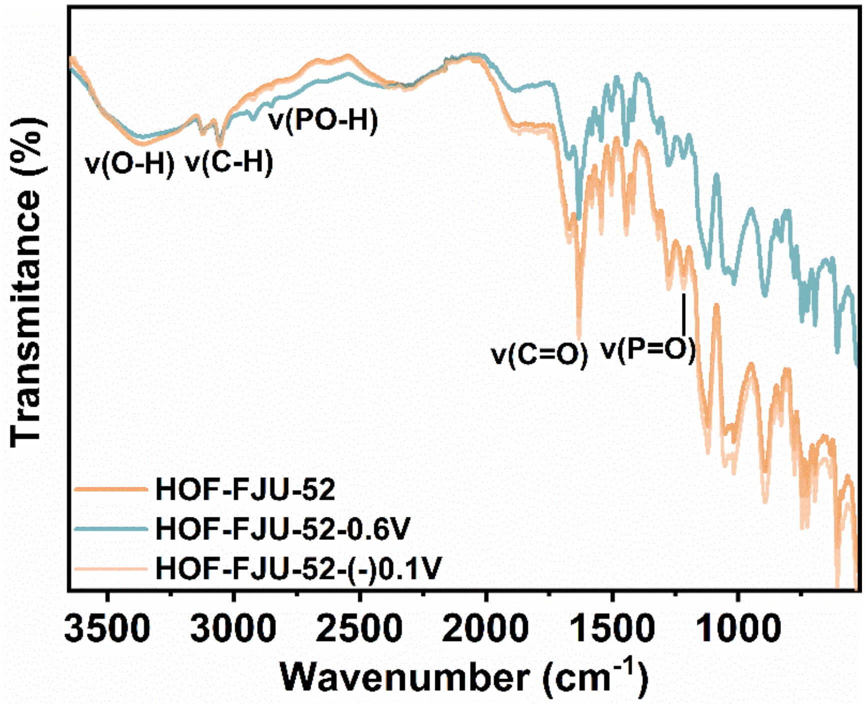


**Supplementary Figure 20.** ATR-IR spectra of eight HOF-FJU-52 single crystals before and after the voltage stimulus at 0.6 V for 20 minutes.

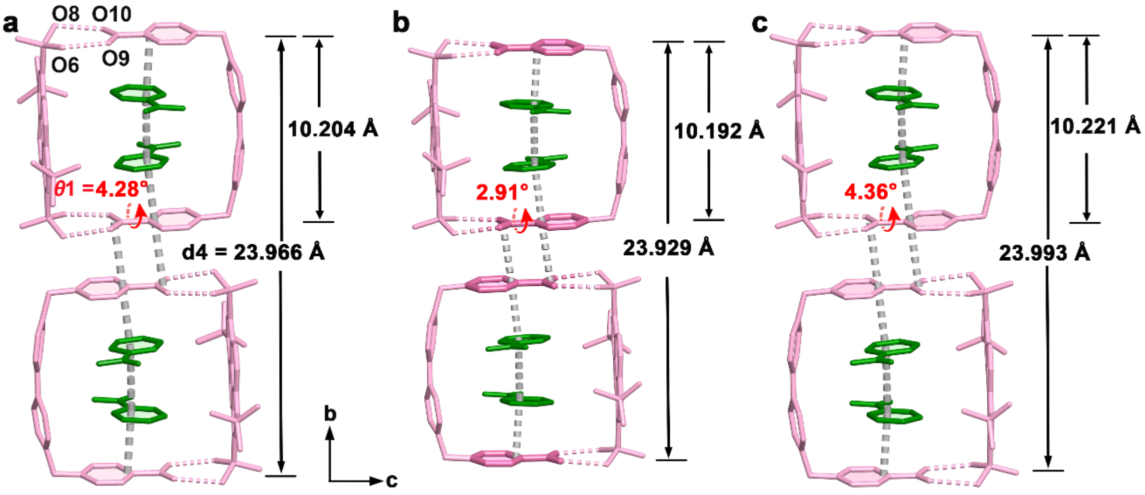


**Supplementary Figure 21.** The changes of dihedral angle and infinite π-π stacking column of HOF-FJU-52 (**a**), HOF-FJU-52-0.6V (**b**) and HOF-FJU-52-(-)0.1V (**c**).

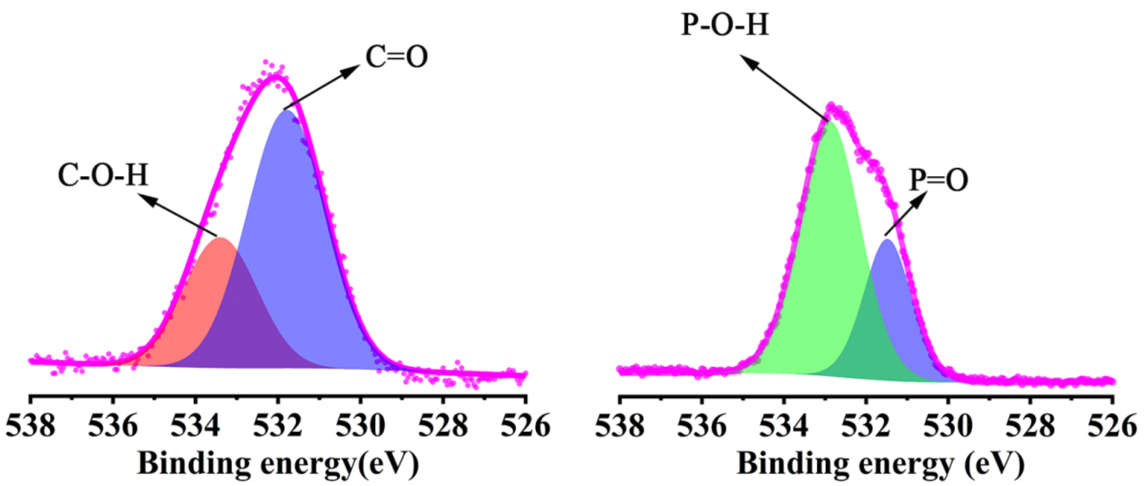


**Supplementary Figure 22.** XPS core-level spectra of O1s of $H_2L$ (left) and $H_8PTP$ (right), respectively.

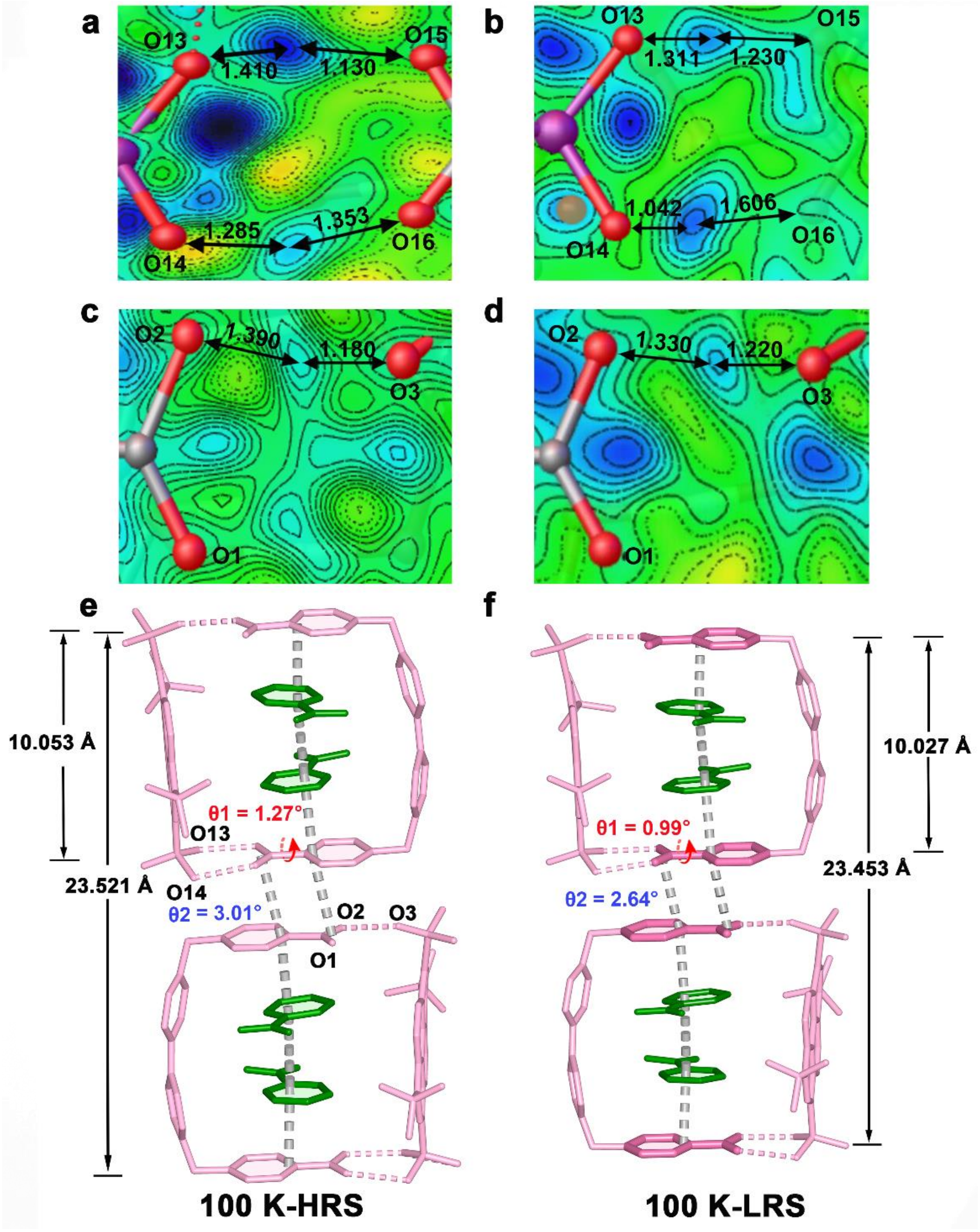


**Supplementary Figure 23.** The structure variations of HOF-FJU-52-100K along the *b* axis under the voltage stimulus. **a-d** The migrations of the electron density peaks between phosphonic and carboxylic acid groups under dc voltages based on the difference Fourier maps. The unit for the distances between electron density peaks and oxygen atoms are given in Å. The changes of dihedral angle and infinite π-π stacking column of HOF-FJU-52-100K-HRS (**e**) and HOF-FJU-52-100K-19V (**f**).

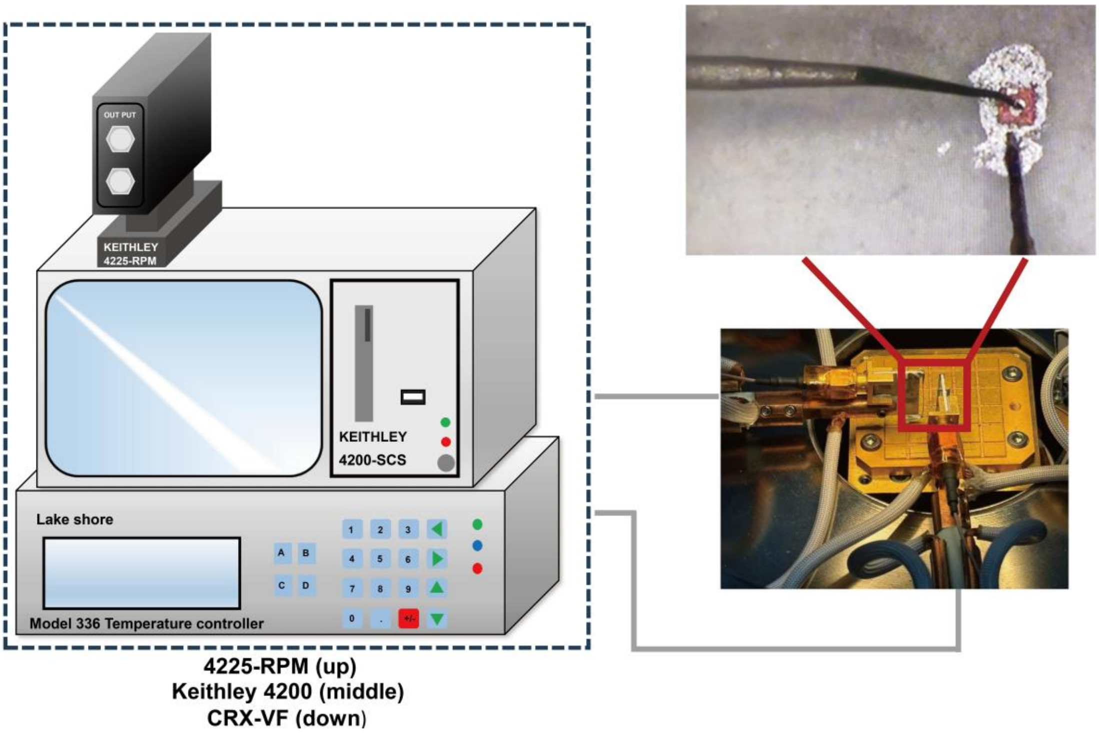


**Supplementary Figure 24.** The microscopic image of the experimental setup.

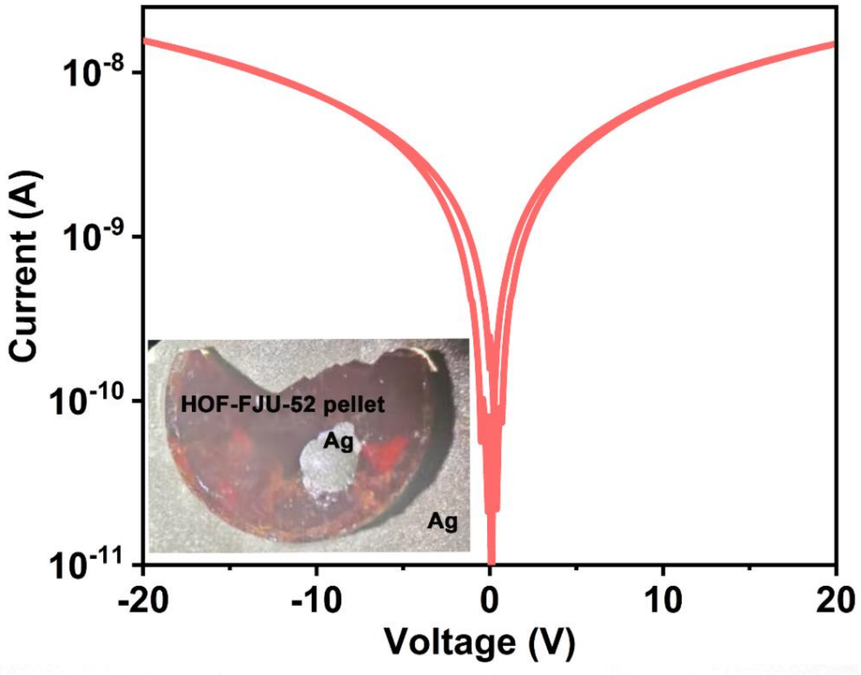


**Supplementary Figure 25.** Electrical performance of the HOF-FJU-52 pellet device. The inset shows a microscopic image of the pellet sample configuration. It is a pity that no obvious RS effect is observed for HOF-FJU-52 pellets samples, though we have tried several times.

**Supplementary Table 1.** Comparison with state of the art MOF/COF-based RS materials.

| Device Structure | $I_{on}$ | $I_{off}$ | Switching Speed (ns) | Switching Voltage (V) | Switching Energy (J) | On/off Ratio | Temperature Stability (K) | Endurance (cycles) | Refs. |
|---|---|---|---|---|---|---|---|---|---|
| Ag/HOF-FJU-52/Ag | 11 pA~ 100 μA | 0.1~1 pA | 34 | ~0.8 | $374\times10^{-21}$ | ~$10^9$ | 500 | $10^6$ | This work |
| Au/$Cu_3(HHTP)_2$/ITO/$SiO_2$ | 1 mA | 1 μA | / | 1 | / | $10^3$ | / | 130 | 1 |
| ITO/polyoxometalate 1/Ag (423K) | 3.9 mA | 143 nA | / | 0.75 | / | 27.3 | 423 | 20 | 2 |
| ITO/Polyoxometalate 1/Ag (543 K) | 0.91 mA | 16.4 nA | / | 0.52 | / | 55.5 | 550 | 20 | 2 |
| Ag/PVA-UiO-66/FTO/glass | 10 μA | 1 nA | / | 0.5 | / | $10^4$ | / | 500 | 3 |
| Au/SAM-SURMOF/Au | 1 μA | ~1 pA | / | 0.7-1 V | / | $10^6$ | / | 10 | 4 |
| Ag/FJU-23-$H_2O$/Ag | 100 μA | 0.1 nA | / | ~0.2 | / | $2\times10^5$ | / | 100 | 5 |
| Ag/ZIF-8 in MeOH vapor/Ag | 1 mA | <0.1 μA | / | 1.80-2.65 | / | ~$10^7$ | / | / | 6 |
| Ag/Rb-CD-MOF/Ag | / | / | $10^9$ | 2 | / | 150 | / | 20–200 | 7 |
| Ag/RSMOF-1/Ag | 50 pA | 1.6 pA | / | 7.5 | / | ~30 | 437 | 50 | 8 |
| Au/HKUST-1/Au | 10 mA | 10 mA | / | ~0.78 | / | 18 | / | $10^7$ | 9 |
| Ag/MIL-53/GaInSn@PDMS | ~1 mA | / | / | 1.2 | / | 200 | / | 200 | 10 |
| rGO/$MoS_2$@ZIF-8/rGO | 5 μA | 0.7 nA | / | 3.3 | / | $7.0\times10^4$ | / | / | 11 |
| Cu/ferrocene@HKUST-1/Au | / | / | / | ~0.6 | / | ~30 | / | / | 12 |
| Al/Zn-TCPP@PVPy/ITO | 30 μA | 10 nA | $10^{10}$ | 0.5 | / | ~$10^3$ | 373 | $10^3$ | 13 |
| ITO/PI-COF film/LiF/Al | 30 nA | 10 mA | / | 2.3 | / | $10^6$ | / | 200 | 14 |
| Ag/COF-5/ITO | / | 1 mA | / | -0.94 | | $10^3$ | | 100 | 15 |
| Ag/COF-TT-BT/ITO | 1 mA | 0.1 μA | / | 1.3 | / | $10^5$ | / | 319 | 16 |
| Ag/$COF_{PBTA+PDA}$/ITO | / | / | / | 1.5 | / | $10^2$ -$10^5$ | 573 | 200 | 17 |

**Supplementary Table 2.** Comparison with state of the art organic RS materials.

| Device Structure | $I_{on}$ | $I_{off}$ | Switching Speed (ns) | Switching Voltage (V) | Switching Energy (J) | On/Off Ratio | Temperature stability (K) | Endurance (cycles) | Refs. |
|---|---|---|---|---|---|---|---|---|---|
| Ag/HOF-FJU-52/Ag | 11pA~ 100 μA | 0.1~ 1 pA | 34 | ~0.8 | $374\times10^{-21}$ | $\sim10^{9}$ | 500 | $10^{6}$ | This work |
| ITO/eCPF-2/Al | 50 mA | 5 μA | / | ~0.6 | / | $\sim10^{4}$ | 573 | 700 | 18 |
| ITO/IPP-V/Au | 1.7 mA | 0.5 mA | $10^{4}$ | 0.5 | / | 2 | / | $10^{6}$ | 19 |
| Ag/SL-2DP/ITO | / | / | / | 0.6 | / | 20 | / | 324 | 20 |
| Cu/poly(1,3,5-trivinyl-1,3,5-trimethyl cyclotrisiloxane) (pV3D3)/Al | 10 μA | 1 nA | 500 | 5 | $2.5\times10^{-11}$ | $10^{7}$ | / | $10^{5}$ | 21 |
| metal/[2]Catenane/Si | / | / | / | ~2 | / | ~4 | 310 | 10 | 22 |
| Al/Ti/Rotaxane /$Al_2O_3$/Al/$SiO_2$/Si Subustrate | / | / | / | -2 | / | 60~80 | / | / | 23 |
| Au/poly[n]rotaxane **1**·4nPF6/Ti/Au | / | / | / | ~1.5 | / | ~7 | / | 20 | 24 |
| **1**·4PF6 (H1)/ ITO | / | / | 60 | ~1.4 | / | 100 | / | / | 25 |
| ITO/DPKAZO 1 or FKAZO 2 /Al | 10 mA | >0.1 μA | / | –1.49/2.61 | / | $10^{2}/10^{4}$ | / | $10^{8}$ | 26 |
| Al/$NI_2$TDPP or $NI_2$TDPP/ITO | 10 mA | 0.1 nA | / | -1.99/-4 | / | $10^{4}/10^{4}$ | / | / | 27 |
| ITO/(MA-H)$_2$(PZDC)·4$H_2O$/Ag | 10 mA | 0.1 μA | | -3 | | $10^{5}$ | | 100 | 28 |
| Au/aniso-TPDAP(100nm)/Au/$SiO_2$/Si | 1 mA | ~10 nA | / | 2.9 | / | $10^{5}$ | 393 | 1000 | 29 |
| Al/Pt-complex (1-PPN)/ITO | 1 mA | ~1 nA | / | 3.4 | / | $10^{5}$ | / | / | 30 |
| Pt/SNACA/Al | ~0.1 A | ~0.1 nA | $10^{10}$ | -2.2 and -2.8 | / | $1/10^{7}/10^{9}$ | / | 100 | 31 |
| ITO/BDOYM/ITO (2-((Z)-2-(4-diphenylamino) benzylidene)-1,2-dihydro-1-oxoinden-3-ylidene) | 2.2 mA | 0.1 μA | / | 1.7 | / | $10^{4}$ | / | / | 32 |
| ITO/TPDBCN/Al (bis{4-[4-[di(ptolyl)amino]phenyl]phenyl}fumaronitrile) | 1 mA | >10 nA | / | ~1.7 | / | $10^{4}$-$10^{6}$ | / | / | 33 |
| Al/AIDCN/Al (2-amino-4,5-imidazoledicarbonitrl) | ~10 μA | >10 nA | 10 | 6 | $6\times10^{-14}$ | $10^{4}$ | / | $10^{6}$ | 34 |
| Au/mer-[Ru(2(phenylazo)pyridine)$_3$](PF$_6$)$_2$/ITO | ~10 μA | ~0.1 nA | ≤30 | ~0.5 | $1.5\times10^{-15}$ | $\sim10^{5}$ | 350 | $10^{12}$ | 35 |
| $AlO_X$/P(VDF-TrFE-CTFE)/ $AlO_X$ | ~0.07μ A | 0.5 nA | / | 4 | / | 100 | / | 2700 | 36 |
| p(g2T-TT) EMIM:TFSI PVDF-HFP | / | / | 20 | 1 | $8\times10^{-14}$ | 10 | 363 | $10^{9}$ | 37 |
| ITO/PI(AAPT-TPA or APT-TPA)/Al | ~100 μA | ~0.1 pA | $10^{3}$ | ~3.5 | / | $10^{8}$-$10^{9}$ | / | $10^{8}$ | 38 |
| Au/(DNA)$_{10}$/Au | 0.1 A | / | $10^{9}$ | 0.73 | / | 30 | / | 100 | 39 |
| ITO/PFOxPy/Al | / | / | / | 2.8 | / | $10^{6}$ | / | / | 40 |
| Al/Au–DT+8HQ+PS/Al | 1 μA | 10 pA | 25 | 2.8 | / | $10^{5}$ | / | 6 | 41 |
| Al/PI (TP6F-PI)/ITO | / | / | $10^{3}$ | 3.2 | / | $10^{5}$ | / | $10^{8}$ | 42 |
| Al/PFT2-Fc/ITO | / | / | $10^{9}$ | -1.9 | / | $10^{3}$ | / | $10^{3}$ | 43 |
| ITO/PVK or PVK-PF (50 nm)/Al | 0.1 μA | 10 pA | / | 2.2 | / | $10^{4}$ | 423 | $10^{8}$ | 44 |
| Si/polyethylenedioxythiophene (PEDT):polystyrene sulphonic acid (PSS)/Si | / | / | $10^{3}$ | 1.5 | / | $\sim10^{4}$ | 473 | / | 45 |
| Au/Co(III) Polymer/Si/$SiO_2$ | 10 pA | 10 nA | $10^{12}$ | -5 | / | $\sim10^{2}$ | / | / | 46 |

**Supplementary Table 3.** Comparison with state of the art inorganic RS materials.

| Device Structure | $I_{on}$ | $I_{off}$ | Switching Speed (ns) | Switching Voltage (V) | Switching Energy (J) | On/off Ratio | Temperature Stability (K) | Endurance (cycles) | Refs. |
|---|---|---|---|---|---|---|---|---|---|
| Ag/HOF-FJU-52/Ag | 11 pA~ 100 µA | 0.1~1 pA | 34 | ~0.8 | $374\times10^{-21}$ | $\sim10^{9}$ | 500 | $10^{6}$ | This work |
| Pt/TiN/$Sb_2Te_3$/GeTe/ TiN/PI | / | / | 60 | 3.5 | $4.8\times10^{-11}$ | 100 | / | $10^{4}$ | 47 |
| Cu/TiW/$Ga_2O_3$/Pt | 10 mA | 0.1 µA | / | 1.3 | / | $10^{5}$ | 473 | 1400 | 48 |
| C-AFM tip/ ZnO nanoisland/$p^{+}$-Si | >1 µA | ~20 pA | / | 0.6-5 | / | $10^{4}$-$10^{8}$ | / | / | 49 |
| TiN/AsTeGeSiN/TiN (OTS) | >100 µA | ~0.1µA | 2 | ~1 | / | $\sim10^{3}$ | 673 | $10^{8}$ | 50 |
| Cu/$SiO_2$/Pt | 500 µA | ~10 pA | / | 0.5~0.8 | / | $\sim10^{7}$ | / | 50 | 51 |
| TiN/Te/TiN/W | 0.5 mA | 0.5 µA | 12 | 0.4 | / | $\sim10^{3}$ | / | $10^{8}$ | 52 |
| Pt/$Cu_2O$/Ag:$Cu_2O$ /$Cu_2O$/Pt | 1 µA | ~1 nA | / | ~0.6 | / | $\sim10^{3}$ | / | / | 53 |
| W/$Cu_2S$/W | 1µA | ~10 pA | / | ~0.3 | / | $\sim10^{5}$ | / | / | 54 |
| Ag/SnS/Pt | ~8µA | ~0.4 pA | 1.5 | ~0.2 | $100\times10^{-15}$ | $\sim10^{8}$ | 425 | $10^{4}$ | 55 |
| TiN/PCH/$SiO_2$ | / | / | 100/20 | 0.8 | $10^{-13}$ | $10^{2}$ | / | $10^{9}$ | 56 |
| Ag/a-Si/p-Si | 0.1 µA | 10 pA | 5 | ~4 | / | $\sim10^{3}$ | / | $10^{6}$ | 57 |
| Pt/$Ta_2O_5$/$HfO_{2-x}$/TiN | 1 µA | 1 nA | / | ~5 | / | $\sim10^{3}$ | 373 | $10^{6}$ | 58 |
| AgTe/TiN/$TiO_2$/Pt | 100 µA | ~1 pA | / | ~0.5 | / | $\sim10^{8}$ | 673 | / | 59 |
| W/NiOy/NbOx/NiOy /W (MIT) | >1 mA | ~0.1 µA | 2 | ~1.4 | / | 5400 | 453 | $10^{8}$ | 60 |
| Pt/$Ta_2O_{5-x}$/$TaO_{2-x}$/Pt | 10 µA | 1 µA | 10 | -1 | / | 10-100 | 523 | $10^{12}$ | 61 |
| Ag/$Cs_3Sb_2Br_9$/Ag | 1 mA | >0.1 µA | $10^{6}$ | ~2.8 | / | $10^{3}$-$10^{4}$ | 420 | 200 | 62 |
| Ag/$(PEA)_2Cs_3Pb_4I_{13}$/ Pt/Ti/$SiO_2$/Si | 1 mA | ~1 pA | / | 0.18 | / | $\sim10^{8}$ | 393 | 230 | 63 |
| Pt/Ag nanodots/$HfO_2$/Pt | >1mA | <1 pA | / | 0.23~0.28 | / | $\sim10^{9}$ | 473 | $10^{8}$ | 64 |
| Au/h-BN/Au | 1 mA | 0.01 pA | 200 | 5 | $8.8\times10^{-21}$ | $10^{11}$ | / | 80000 | 65 |
| Pt/MgO:Ag/Pt, Pt/SiOxNy:Ag/Pt/, Pt/HfOx:Ag/Pt | 1 µA, 100 µA, 1 mA | 0.1 pA | 500 | ~0.3, ~0.3 ~0.2 | $7.5\times10^{-11}$ | $3.3\times10^{3}$, $10^{5}$, $10^{10}$ | 600 | $10^{6}$ | 66 |
| Au/MCA−BP/Au (organic–inorganic hybrid memristor) | 0.1 µA | 10 pA | $10^{6}$ | 4 | / | $10^{4}$ | / | / | 67 |

**Supplementary Table 4.** Crystal data and structure refinement for HOF-FJU-52 under voltage sweeping and different temperatures stimulus (R: the recovered sample).

| **Compounds** | **HOF-FJU-52** | **HOF-FJU-52-0.6V** | **HOF-FJU-52--(-)0.1V** | **HOF-FJU-52-250K** | **HOF-FJU-52-200K** | **HOF-FJU-52-150K** | **HOF-FJU-52-100K** | **HOF-FJU-52-150K-R -** | **HOF-FJU-52-200K-R** | **HOF-FJU-52-250K-R** | **HOF-FJU-52-293K-R** |
|---|---|---|---|---|---|---|---|---|---|---|---|
| CCDC# | 2052476 | 2058177 | 2058181 | 2095208 | 2095212 | 2105287 | 2105289 | 2225349 | 2225356 | 2225743 | 2225358 |
| Empirical formula | $C_{68}H_{78}N_4O_{32}P_4$ | $C_{68}H_{78}N_4O_{32}P_4$ | $C_{68}H_{78}N_4O_{32}P_4$ | $C_{68}H_{78}N_4O_{32}P_4$ | $C_{68}H_{79}N_4O_{32.5}P_4$ | $C_{68}H_{79}N_4O_{32.5}P_4$ | $C_{68}H_{79}N_4O_{32.5}P_4$ | $C_{68}H_{79}N_4O_{325}P_4$ | $C_{68}H_{79}N_4O_{32.5}P_4$ | $C_{68}H_{79}N_4O_{32.5}P_4$ | $C_{68}H_{78}N_4O_{32}P_4$ |
| Formula weight | 1587.22 | 1587.22 | 1587.22 | 1587.22 | 1596.23 | 1596.23 | 1596.23 | 1596.23 | 1596.23 | 1596.23 | 1587.22 |
| Temperature/K | 293 (13) | 294.0(6) | 293.8(2) | 250.00(10) | 200.00(10) | 150.00(10) | 100 | 150.00(10) | 200.00(10) | 250.00(2) | 292.8(8) |
| Crystal system | orthorhombic | orthorhombic | orthorhombic | orthorhombic | orthorhombic | orthorhombic | orthorhombic | orthorhombic | orthorhombic | orthorhombic | orthorhombic |
| Space group | *Ccce* | *Ccce* | *Ccce* | *Ccce* | *C2cb* | *C2cb* | *C2cb* | *C2cb* | *C2cb* | *C2cb* | *Ccce* |
| $a$/Å | 18.7971(8) | 18.7672(12) | 18.7774(8) | 18.7672(7) | 18.7340(3) | 18.7232(2) | 18.7325(3) | 18.7470(3) | 18.7451(4) | 18.7532(4) | 18.7750(4) |
| $b$/Å | 27.3966(11) | 27.352(2) | 27.4144(18) | 27.3380(14) | 26.8871(4) | 26.8414(4) | 26.8159(5) | 26.8493(4) | 26.8826(6) | 26.9953(10) | 27.3660(7) |
| $c$/Å | 28.1438(10) | 28.109(2) | 28.1723(18) | 28.3004(11) | 28.2510(5) | 28.2465(4) | 28.2322(5) | 28.2455(4) | 28.2665(7) | 28.2453(9) | 28.1988(7) |
| $\alpha$/° | 90 | 90 | 90 | 90 | 90 | 90 | 90 | 90 | 90 | 90 | 90 |
| $\beta$/° | 90 | 90 | 90 | 90 | 90 | 90 | 90 | 90 | 90 | 90 | 90 |
| $\gamma$/° | 90 | 90 | 90 | 90 | 90 | 90 | 90 | 90 | 90 | 90 | 90 |
| Volume/Å$^3$ | 14493.4(10) | 14429(2) | 14502.3(15) | 14519.7(11) | 14230.1(4) | 14195.5(3) | 14181.9(4) | 14217.2(4) | 14244.0(6) | 14299.1(8) | 14488.4(6) |
| $Z$ | 8 | 8 | 8 | 8 | 8 | 8 | 8 | 8 | 8 | 8 | 8 |
| $\rho_{calc}$g/cm$^3$ | 1.455 | 1.461 | 1.454 | 1.452 | 1.49 | 1.494 | 1.495 | 1.491 | 1.489 | 1.483 | 1.455 |
| μ/mm$^{-1}$ | 1.772 | 1.780 | 1.771 | 1.769 | 1.813 | 1.818 | 1.82 | 1.815 | 1.812 | 1.805 | 1.773 |
| F(000) | 6640 | 6640 | 6640 | 6640 | 6680 | 6680 | 6680 | 6680 | 6680 | 6680 | 6640 |
| Radiation | CuKα (λ = 1.54184) | CuKα (λ = 1.54184) | CuKα (λ = 1.54184) | CuKα (λ = 1.54184) | CuKα (λ = 1.54184) | CuKα (λ = 1.54184) | CuKα (λ = 1.54184) | CuKα (λ = 1.54184) | CuKα (λ = 1.54184) | CuKα (λ = 1.54184) | CuKα (λ = 1.54184) |
| Reflections collected | 26786 | 24175 | 26051 | 25844 | 27096 | 27278 | 26885 | 26302 | 25985 | 25506 | 23813 |
| Independent reflections | 6403 [$R_{int}$ = 0.0580, $R_{sigma}$ = 0.0345] | 6334 [$R_{int}$ = 0.0736, $R_{sigma}$ = 0.0467] | 6409 [$R_{int}$ = 0.0899, $R_{sigma}$ = 0.0502] | 6966 [$R_{int}$ = 0.0596, $R_{sigma}$ = 0.0855] | 10863 [$R_{int}$ = 0.0286, $R_{sigma}$ = 0.0304] | 10658 [$R_{int}$ = 0.0300, $R_{sigma}$ = 0.0306] | 11013 [$R_{int}$ = 0.0370, $R_{sigma}$ = 0.0405] | 11078 [$R_{int}$ = 0.0275, $R_{sigma}$ = 0.0294] | 11735 [$R_{int}$ = 0.0445, $R_{sigma}$ = 0.0550] | 10789 [$R_{int}$ = 0.0576, $R_{sigma}$ = 0.0665] | 7101 [Rint = 0.0326, Rsigma = 0.0201] |
| Data/restraints/parameters | 6403/74/538 | 6334/62/528 | 6409/38/507 | 6966/56/518 | 10863/84/1012 | 10658/114/1044 | 11013/101/1014 | 11078/79/1007 | 11735/91/1008 | 10792/69/1006 | 7101/37/521 |
| Goodness-of-fit on $F^2$ | 1.027 | 1.114 | 1.161 | 1.154 | 1.046 | 1.036 | 1.033 | 1.059 | 1.032 | 1.037 | 0.926 |
| Final $R$ indexes [$I$>=2σ ($I$)][(a)] | $R_1$ = 0.0875, $wR_2$ = 0.2499 | $R_1$ = 0.0894, $wR_2$ = 0.2246 | $R_1$ = 0.0932, $wR_2$ = 0.2456 | $R_1$ = 0.1172, $wR_2$ = 0.2233 | $R_1$ = 0.0676, $wR_2$ = 0.1852 | $R_1$ = 0.0681, $wR_2$ = 0.1827 | $R_1$ = 0.0819, $wR_2$ = 0.2212 | $R_1$ = 0.0717, $wR_2$ = 0.1935 | $R_1$ = 0.0765, $wR_2$ = 0.2022 | $R_1$ = 0.0876, $wR_2$ = 0.2369 | $R_1$ = 0.0897, $wR_2$ = 0.2694 |
| Final $R$ indexes [all data][(a)] | $R_1$ = 0.1312, $wR_2$ = 0.3028 | $R_1$ = 0.1707, $wR_2$ = 0.2798 | $R_1$ = 0.1538, $wR_2$ = 0.2926 | $R_1$ = 0.1926, $wR_2$ = 0.2489 | $R_1$ = 0.0761, $wR_2$ = 0.1963 | $R_1$ = 0.0757, $wR_2$ = 0.1919 | $R_1$ = 0.0922, $wR_2$ = 0.2334 | $R_1$ = 0.0766, $wR_2$ = 0.2014 | $R_1$ = 0.0945, $wR_2$ = 0.2194 | $R_1$ = 0.1433, $wR_2$ = 0.3049 | $R_1$ = 0.1137, $wR_2$ = 0.3021 |
| Largest diff. peak/hole/e Å$^{-3}$ | 0.46/-0.38 | 0.50/-0.27 | 0.85/-0.38 | 0.38/-0.34 | 0.81/-0.49 | 0.95/-0.47 | 0.95/-0.52 | 0.98/-0.49 | 0.83/-0.58 | 0.83/-0.55 | 0.72/-0.49 |

$(a)\ R_1=\sum||F_o|-|F_c||/\sum|F_o|;\ wR_2=[\sum w(|F_o|^2-|F_c|^2)^2/\sum w(F_o{}^2)^2]^{1/2}$

**Supplementary Table 5.** Crystal data and structure refinement for HOF-FJU-52-100K under voltage sweeping.

| **Compounds** | **HOF-FJU-52-100K-HRS** | **HOF-FJU-52-100K-19V** |
|---|---|---|
| CCDC# | 2246710 | 2246372 |
| Empirical formula | $C_{68}H_{78}N_4O_{32.5}P_4$ | $C_{68}H_{78}N_4O_{32.5}P_4$ |
| Formula weight | 1595.22 | 1595.22 |
| Temperature/K | 100 | 100 |
| Crystal system | orthorhombic | orthorhombic |
| Space group | *C2cb* | *C2cb* |
| $a$/Å | 18.7533(3) | 18.7283(3) |
| $b$/Å | 26.8620(7) | 26.7823(6) |
| $c$/Å | 28.3160(5) | 28.2719(6) |
| $\alpha$/° | 90 | 90 |
| $\beta$/° | 90 | 90 |
| $\gamma$/° | 90 | 90 |
| Volume/Å$^3$ | 14264.2(5) | 14180.8(5) |
| $Z$ | 8 | 8 |
| $\rho_{calc}$g/cm$^3$ | 1.486 | 1.494 |
| μ/mm$^{-1}$ | 1.809 | 1.82 |
| F(000) | 6672 | 6672 |
| Radiation | CuKα (λ = 1.54184) | CuKα (λ = 1.54184) |
| Reflections collected | 19680 | 20601 |
| Data/restraints/parameters | 10440/21/994 | 10159/10/994 |
| Goodness-of-fit on $F^2$ | 1.019 | 1.039 |
| Final $R$ indexes [$I$>=2σ ($I$)][(a)] | $R_1$ = 0.0928, $wR_2$ = 0.2384 | $R_1$ = 0.0752, $wR_2$ = 0.1973 |
| Final $R$ indexes [all data][(a)] | $R_1$ = 0.1076, $wR_2$ = 0.2604 | $R_1$ = 0.0835, $wR_2$ = 0.2111 |
| Largest diff. peak/hole/e Å$^{-3}$ | 0.98/-0.63 | 1.14/-0.51 |

(*a*) $R_1=\sum||F_o|-|F_c||/\sum|F_o|$; $wR_2=[\sum w(|F_o|^2-|F_c|^2)^2/\sum w(F_o^2)^2]^{1/2}$

## Supplementary References


1. Liu, L. et al. High-quality two-dimensional metal-organic framework nanofilms for nonvolatile memristive switching. *Small Struct.* **2**, 2000077 (2021).
2. Huang, Y.-R. et al. Thermal-responsive polyoxometalate–metalloviologen hybrid: Reversible intermolecular three-component reaction and temperature-regulated resistive switching behaviors. *Angew. Chem. Int. Ed.* **60**, 16911-16916 (2021).
3. Nhu Hoang Tran, T. et al. C-AFM study on multi-resistive switching modes observed in metal-organic frameworks thin films. *Org. Electron.* **93**, 106136 (2021).
4. Albano, L. G. S. et al. Ambipolar resistive switching in an ultrathin surface-supported metal–organic framework vertical heterojunction. *Nano Lett.* **20**, 1080-1088 (2020).
5. Yao, Z. et al. Simultaneous implementation of resistive switching and rectifying effects in a metal-organic framework with switched hydrogen bond pathway. *Sci. Adv.* **5**, eaaw4515 (2019).
6. Liu, Y. et al. Alcohol-mediated resistance-switching behavior in metal-organic framework-based electronic devices. *Angew. Chem. Int. Ed.* **55**, 8884-8888 (2016).
7. Yoon, S. M., Warren, S. C. & Grzybowski, B. A. Storage of electrical information in metal-organic-framework memristors. *Angew. Chem. Int. Ed.* **53**, 4437-4441 (2014).
8. Pan, L. et al. A resistance-switchable and ferroelectric metal-organic framework. *J. Am. Chem. Soc.* **136**, 17477-17483 (2014).
9. Pan, L. et al. Metal-organic framework nanofilm for mechanically flexible information storage applications. *Adv. Funct. Mater.* **25**, 2677-2685 (2015).
10. Yi, X. et al. Intrinsically stretchable resistive switching memory enabled by combining a liquid metal-based soft electrode and a metal-organic framework insulator. *Adv. Electron. Mater.* **5**, 1800655 (2019).
11. Huang, X. et al. Coating two-dimensional nanomaterials with metal–organic frameworks. *ACS nano* **8**, 8695-8701 (2014).
12. Wang, Z. et al. Resistive switching nanodevices based on metal-organic frameworks. *ChemNanoMat* **2**, 67-73 (2016).
13. Ding, G. et al. 2D metal-organic framework nanosheets with time-dependent and multilevel memristive switching. *Adv. Funct. Mater.* **29**, 1806637 (2019).
14. Sun, B. et al. Resistive switching memory performance of two-dimensional polyimide covalent organic framework films. *ACS Appl. Mater. Interfaces* **12**, 51837-51845 (2020).
15. Li, T. et al. 2D oriented covalent organic frameworks for alcohol-sensory synapses. *Mater. Horiz.* 8, 2041-2049 (2021).
16. Li, C. et al. Towards high-performance resistive switching behavior through embedding a D-A system into 2D imine-linked covalent organic frameworks. *Angew. Chem. Int. Ed.* **60**, 27135-27143 (2021).
17. Liu, J. et al. A robust nonvolatile resistive memory device based on a freestanding ultrathin 2D imine polymer film. *Adv. Mater.* **31**, 1902264 (2019).
18. Tao, Y. et al. Electrochemical preparation of porous organic polymer films for high-performance memristors. *Angew. Chem. Int. Ed.* **61**, e202209952 (2022).
19. Wang, L. et al. Viologen-hypercrosslinked ionic porous polymer films as active layers for electronic and energy storage devices. *Adv. Mater. Interfaces* **5**, 1701679 (2018).
20. Liu, L. et al. A highly crystalline single layer 2D polymer for low variability and excellent scalability molecular memristors. *Adv. Mater.* **35**, 2208377 (2023).
21. Jang, B. C. et al. Flexible nonvolatile polymer memory array on plastic substrate via initiated chemical vapor deposition. *ACS Appl. Mater. Interfaces* **8**, 12951-12958 (2016).
22. Collier, C. P. et al. A [2]catenane-based solid state electronically reconfigurable switch. *Science* **289**, 1172-1175 (2000).
23. Collier, C. P. et al. Electronically configurable molecular-based logic gates. *Science* **285**, 391 (1999).

24. Zhang, W. et al. A solid-state switch containing an electrochemically switchable bistable poly[n]rotaxane. *J. Mater. Chem.* **21**, 1487-1495 (2011).
25. Feng, M. et al. Stable, reproducible nanorecording on rotaxane thin films. *J. Am. Chem. Soc.* **127**, 15338-15339 (2005).
26. Miao, S. et al. Tailoring of molecular planarity to reduce charge injection barrier for high-performance small-molecule-based ternary memory device with low threshold voltage. *Adv. Mater.* **24**, 6210-6215 (2012).
27. Li, Y. et al. Toward highly robust nonvolatile multilevel memory by fine tuning of the nanostructural crystalline solid-state order. *Small* **17**, 2100102 (2021).
28. Liu, Q. et al. Reversible photo/thermal stimuli-responsive electrical bistability performance in supramolecular co-crystals accompanied by crystalline-to-amorphous transformations. *J. Mater. Chem. C* **8**, 3258-3267 (2020).
29. Kim, J. et al. Control of anisotropy of a redox-active molecule-based film leads to non-volatile resistive switching memory. *Chem. Sci.* **10**, 10888-10893 (2019).
30. Li, Y. et al. Supramolecular self-assembly and dual-switch vapochromic, vapoluminescent, and resistive memory behaviors of amphiphilic platinum (II) complexes. *J. Am. Chem. Soc.* **139**, 13858-13866 (2017).
31. Li, H. et al. A small-molecule-based device for data storage and electro-optical switch applications. *J. Mater. Chem.* **21**, 5860 (2011).
32. Shang, Y. et al. A triphenylamine-containing donor−acceptor molecule for stable, reversible, ultrahigh density data storage. *J. Am. Chem. Soc.* **129**, 11674-11675 (2007).
33. Ma, Y. et al. Improving the on/off ratio and reversibility of recording by rational structural arrangement of donor-acceptor molecules. *Adv. Funct. Mater.* **20**, 803-810 (2010).
34. Ma, L. P., Liu, J. & Yang, Y. Organic electrical bistable devices and rewritable memory cells. *Appl. Phys. Lett.* **80**, 2997-2999 (2002).
35. Goswami, S. et al. Robust resistive memory devices using solution-processable metal-coordinated azo aromatics. *Nat. Mater.* **16**, 1216-1224 (2017).
36. Xu, T. et al. Excellent low-voltage operating flexible ferroelectric organic transistor nonvolatile memory with a sandwiching ultrathin ferroelectric film. *Sci. Rep.* **7**, 8890 (2017).
37. Melianas, A. et al. Temperature-resilient solid-state organic artificial synapses for neuromorphic computing. *Sci. Adv.* ***6***, eabb2958 (2019).
38. Kuorosawa, T., Chueh, C.-C., Liu, C.-L., Higashihara, T., Ueda, M., Chen, W.-C. High performance volatile polymeric memory devices based on novel triphenylamine-based polyimides containing mono- or dual-mediated phenoxy linkages. *Macromolecules* **43**, 1236-1244 (2010).
39. Qin, S., Dong, R., Yan, X. & Du, Q. A reproducible write–$(read)_n$–erase and multilevel bio-memristor based on DNA molecule. *Org. Electron.* **22**, 147-153 (2015).
40. Ling, Q.-D. et al. A dynamic random access memory based on a conjugated copolymer containing electron-donor and -acceptor moieties. *Angew. Chem. Int. Ed.* **45**, 2947-2951 (2006).
41. Ouyang, J. et al. Programmable polymer thin film and non-volatile memory device. *Nat. Mater.* **3**, 918-922 (2004).
42. Ling, Q.-D. et al. Synthesis and dynamic random access memory behavior of a functional polyimide. *J. Am. Chem. Soc.* **128**, 8732-8733 (2006).
43. Choi, T.-L. et al. Synthesis and nonvolatile memory behavior of redox-active conjugated polymer-containing ferrocene. *J. Am. Chem. Soc.* **129**, 9842-9843 (2007).
44. Xie, L.-H., Ling, Q.-D., Hou, X.-Y. & Huang, W. An effective friedel−crafts postfunctionalization of poly(n-vinylcarbazole) to tune carrier transportation of supramolecular organic semiconductors based on π-stacked polymers for nonvolatile flash memory cell. *J. Am. Chem. Soc.* **130**, 2120-2121 (2008).
45. Möller, S. et al. A polymer/semiconductor write-once read-many-times memory. *Nature* **426**, 166–169 (2003).

46. Bandyopadhyay, A., Sahu, S. & Higuchi, M. Tuning of nonvolatile bipolar memristive switching in Co (III) polymer with an extended azo aromatic ligand. *J. Am. Chem. Soc.* **133**, 1168-1171 (2011).
47. Khan, A. I. et al. Ultralow–switching current density multilevel phase-change memory on a flexible substrate. *Science* **373**, 1243-1247 (2021).
48. Gan, K.-J. et al. Highly durable and flexible gallium-based oxide conductive-bridging random access memory. *Sci. Rep.* **9**, 14141 (2019).
49. Qi, J. et al. Resistive switching in single epitaxial ZnO nanoislands. *ACS nano* **6**, 1051-1058 (2012).
50. Lee, M. J. et al. A plasma-treated chalcogenide switch device for stackable scalable 3D nanoscale memory. *Nat. Commun.* **4**, 2629 (2013).
51. Chen, W., Barnaby, H. J. & Kozicki, M. N. Volatile and non-volatile switching in Cu-$SiO_2$ programmable metallization cells. *IEEE Electron Device Lett* **37**, 580-583 (2016).
52. Shen, J. et al. Elemental electrical switch enabling phase segregation–free operation. *Science* **374**, 1390-1394 (2021).
53. Song, J. et al. Bidirectional threshold switching in engineered multilayer ($Cu_2O$/Ag:$Cu_2O$/$Cu_2O$) stack for cross-point selector application. *Appl. Phys. Lett.* **107**, 113504 (2015).
54. Lim, S. et al. CMOS compatible low-power volatile atomic switch for steep-slope FET devices. *Appl. Phys. Lett.* **113**, 033501 (2018).
55. Lu, X. F. et al. Exploring low power and ultrafast memristor on p-type van der waals SnS. *Nano Lett.* **21**, 8800-8807 (2021).
56. Ding, K. et al. Phase-change heterostructure enables ultralow noise and drift for memory operation. *Science* **366**, 210-215 (2019).
57. Jo, S. H. & Lu, W. CMOS compatible nanoscale nonvolatile resistance switching memory. *Nano Lett.* **8**, 392-397 (2008).
58. Yoon, J. H. et al. Highly uniform, electroforming-free, and self-rectifying resistive memory in the Pt/$Ta_2O_5$/$HfO_{2-x}$/TIN structure. *Adv. Funct. Mater.* **24**, 5086-5095 (2014).
59. Song, J. et al. Effects of liner thickness on the reliability of AgTe/$TiO_2$-based threshold switching devices. *IEEE Trans on Electron Devices* **64**, 4763-4767 (2017)..
60. Park, J. et al. Multi-layered NiOy/NbOx/NiOy fast drift-free threshold switch with high $I_{on}/I_{off}$ ratio for selector application. *Sci. Rep.* **7**, 4068 (2017).
61. Lee, M.-J. et al. A fast, high-endurance and scalable non-volatile memory device made from asymmetric $Ta_2O_{5-x}$/$TaO_{2-x}$ bilayer structures. *Nat. Mater.* **10**, 625-630 (2011).
62. Mao, J.-Y. et al. Lead-free monocrystalline perovskite resistive switching device for temporal information processing. *Nano Energy* **71**, 104616 (2020).
63. Kim, H. et al. Quasi-2D halide perovskites for resistive switching devices with on/off ratios above $10^9$. *NPG Asia Mater.* **12**, 1-11(2020).
64. Hua, Q. et al. A threshold switching selector based on highly ordered Ag nanodots for X-point memory applications. *Adv. Sci.* **6**, 1900024 (2019).
65. Chen, S. et al. Wafer-scale integration of two-dimensional materials in high-density memristive crossbar arrays for artificial neural networks. *Nat. Electron.* **3**, 638-645 (2020).
66. Wang, Z. et al. Memristors with diffusive dynamics as synaptic emulators for neuromorphic computing. *Nat. Mater.* **16**, 101-108 (2017).
67. Hsieh, Y.-L. & Su, C.-Y. Black phosphorus nanosheet/melamine cyanurate assemblies as functional active layers for artificial synapse memristors. *ACS Appl. Nano Mater.* **4**, 9584-9594 (2021).
